# Construction of thickness-controllable bimetallic sulfides/reduced graphene oxide as a binder-free positive electrode for hybrid supercapacitors†

**DOI:** 10.1039/d3ra05326a

**Published:** 2023-10-05

**Authors:** Ramage M. Ghanem, Doaa A. Kospa, Awad I. Ahmed, Amr Awad Ibrahim, Ahmed Gebreil

**Affiliations:** a Department of Chemistry, Faculty of Science, Mansoura University Al-Mansoura 35516 Egypt amr_awad@mans.edu.eg; b Nile Higher Institutes of Engineering and Technology El-Mansoura Egypt

## Abstract

Devices for electrochemical energy storage with exceptional capacitance and rate performance, outstanding energy density, simple fabrication, long-term stability, and remarkable reversibility have always been in high demand. Herein, a high-performance binder-free electrode (3D NiCuS/rGO) was fabricated as a supercapacitor by a simple electrodeposition process on a Ni foam (NF) surface. The thickness of the deposited materials on the NF surface was adjusted by applying a low cycle number of cyclic voltammetry (5 cycles) which produced a thin layer and thus enabled the easier penetration of electrolytes to promote electron and charge transfer. The NiCuS was anchored by graphene layers producing nicely integrated materials leading to a higher electroconductivity and a larger surface area electrode. The as-fabricated electrode displayed a high specific capacitance (2211.029 F g^−1^ at 5 mV s^−1^). The NiCuS/rGO/NF//active carbon device can achieve a stable voltage window of 1.5 V with a highly specific capacitance of 84.3 F g^−1^ at a current density of 1 A g^−1^. At a power density of 749 W kg^−1^, a satisfactory energy density of 26.3 W h kg^−1^ was achieved, with outstanding coulombic efficiency of 100% and an admirable life span of 96.2% after 10 000 GCD cycles suggesting the significant potential of the as-prepared materials for practical supercapacitors.

## Abstract 2

Devices for electrochemical energy storage with exceptional capacitance and rate performance, outstanding energy density, simple fabrication, long-term stability, and remarkable reversibility have always been in high demand.

## Graphical abstract

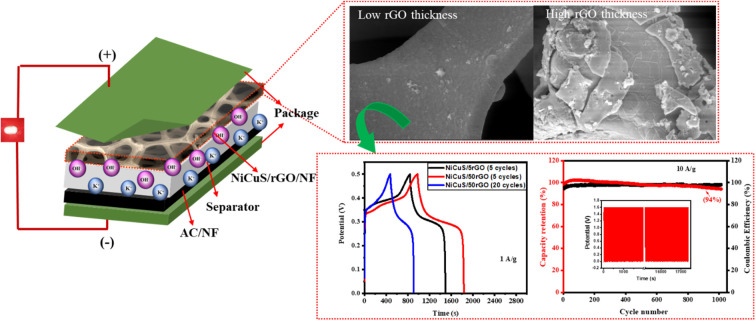

## 1. Introduction

The production and consumption of energy that depends on the burning of fossil fuels will have a substantial influence on both the global economy and ecology.^1–3^ As a result, environmentally friendly sources of energy like solar, wind, and hydro energy must be developed for converting and storing energy.^3^ However, supercapacitors and batteries are more reliable and effective in this regard.^4–6^ Based on the electrochemical energy conversion meaning, non-conventional energy sources have been utilized in portable electronic devices including supercapacitors (SCs), fuel cells (FCs), solar cells, and batteries.^7,8^ Fuel cells use electrochemical processes with an oxidant (typically oxygen) to transform chemical energy from an external fuel like hydrocarbons, hydrogen, or alcohol into electrical energy.^9^ Although batteries have high specific energy and are widely used in a variety of electrical applications, they retain energy by charge storage through an electrochemical reaction.^10,11^ Nonetheless, their actual application was inhibited by a variety of factors such as liquid electrolyte loss, volatility, flammability, poor power densities, and thermal instability.^12,13^ Otherwise, supercapacitors are newly fabricated devices between the battery and conventional capacitors that have attracted substantial interest in many fields in recent years.^14–17^ The hybrid supercapacitor (HSC), which can maintain a high power density while increasing energy density, can significantly broaden the practical application of SCs.^18,19^ Various charge storage strategies of the cathodes and anodes can adjust the equilibrium of power and energy densities, which enhances the efficiency of supercapacitors.^20^

Supercapacitors are categorized according to the kinds of electrode materials they use as electric double-layer capacitors (EDLCs), pseudocapacitors, and hybrid supercapacitors of EDLC and pseudocapacitors. Large power density, high conductivity, and enduring stability are all features of EDLCs, however, their low specific capacitance may limit their applications to some extent.^20–22^ Although pseudo-capacitors have better specific capacitance than EDLC-based materials, they suffer multiple challenges including low power densities and cyclic stability.^23^ Recent studies have shown that the HSC is known to be the best potential candidate for achieving high-performance energy storage systems.^24,25^ The combination of widely utilized EDLCs and pseudocapacitor materials is projected to enhance the HSCs' energy densities.^26,27^

Another major point is finding a way to combine high capacitance with high power density, which is the major benefit of supercapacitors.^28^ One of the fundamental objectives of enhancing the supercapacitor's efficiency is to find working electrodes that preserve both high-power density and specific capacitance. Numerous carbon-based electrodes including graphene oxide (GO), carbon nanotubes (CNTs), carbon-derived metal–organic frameworks (MOFs), heteroatom-doped carbons and activated carbon aerogel were utilized as EDLCs.^29–31^ While metal sulfides, metal oxides, metal phosphides, and conducting polymers (CPs) were used as pseudo-capacitor.^20^ Lately, transition-metal sulfides (TMS) have been effectively used as positive electrodes in several electrochemical applications for their superior pseudocapacity performance including synergistically high electrical conductivity, reversible capacity, and mechanical and thermal stability than those of their matching metal oxides. Meanwhile, different metal sulfides such as NiS,^32^ MoS_2_,^33^ ZnS,^34^ MnS_2_,^35^ SnS_2_,^36^ and CuS^37^ have been utilized as capable pseudo-capacitor electrodes. Among all these materials, NiS and CuS have received the most attention because of their higher redox reactions, greater reversible capacity, high electrical conductivity, simplicity of manufacturing, and low toxicity.^38,39^ However, due to their semiconducting behaviour and significant volume change through the galvanostatic charge–discharge (GCD) measurements, CuS is uncommon for supercapacitors.^40,41^ The mean drawback of NiS electrodes in supercapacitors is their weak cycling abilities due to the electrical contact loss between the active substances and the current collector.^42,43^ Hence, nickel–copper sulfides (NiCuS) are predicted to provide outstanding capacitance and long-term stable cycling capabilities, to solve the drawbacks of these materials.^44^ The enhanced performance of NiCuS outperforms binary metal sulfide (NiS or CuS) for some reasons: the higher redox reaction and multiple oxidation states of the optimized bimetallic composition, the improved conductivity with low resistance, and the increased specific active sites with connected nanosheets.^45,46^ A hybrid supercapacitor can also be made by merging three-dimensional (3D) graphene oxide with metal sulfide electrodes to increase their energy density. GO structure can enhance the electrochemical performances and the cycling stabilities of electrodes because these structures provide both connected graphene networks for strong electric conductivity and 3D porous channels for rapid charge transfer.^47^ Additionally, 3D GO provides a larger positive side potential in the neutral electrolyte, more electroactive sites, and multiple oxidation states.^48^ The electrode materials design has a major effect on the supercapacitor performance.^49^ Metal chalcogenide thin films have been formed using several techniques including chemical vapour deposition, electrodeposition, anodization, sequential ionic adsorption, and hydrothermal procedures.^19^ In comparison to alternative procedures that are difficult, time-consuming, and energy-intensive, electrodeposition may be a very easy and inexpensive method for depositing metal sulfide layers on large and complex surfaces.

To get over the above restrictions, we created a simple and environmentally friendly one-step electrochemical deposition process to synthesize a 3D structured NiCuS/rGO array on the NF surface for hybrid SC. The NiCuS sheets were entirely and properly anchored to the reduced graphene oxide (rGO) which was reduced through the electrodeposition process, according to structural and morphological characterization. The electrode was deposited on the NF surface as a current collector with different cyclic voltammetry numbers to control the thickness of the membrane on the NF surface. The electrodes employed as positive electrodes in a 3–3-configuration cell reached an incredibly ultrahigh capacity of 920 C g^−1^ at the applied current density of 1 A g^−1^ (2211.029 F g^−1^ at 5 mV s^−1^) for NiCuS/50rGO (5 cycles). Finally, the asymmetric supercapacitor device of NiCuS/rGO/NF interconnected sheets and active carbon/NF as a negatively charged electrode (cathode) was constructed and tested to produce satisfactory energy and power densities of 26.3 W h kg^−1^ and 749 W kg^−1^, respectively through the applied current density of 1 A g^−1^.

## 2. Experimental section

### 2.1. Materials

Without any additional pre-treatments, all of the reagents and analytical-grade chemicals were used. Hydrochloric acid (HCl; 36%), potassium permanganate (KMnO_4_), ethanol (C_2_H_6_O; 99%), sulfuric acid (H_2_SO_4_; 98%), hydrogen peroxide (H_2_O_2_; 30%), and thiourea (CH_4_N_2_S) were provided from Merck. Moreover, nickel chloride (NiCl_2_·6H_2_O), sodium nitrate (NaNO_3_), copper chloride (CuCl_2_), *N*-methyl-2-pyrrolidone, potassium hydroxide (KOH), graphite (99.999%), nickel foam (Ni-foam), acetylene black, and polyvinylidene fluoride were got from Sigma Aldrich. Commercial active carbon was used in the cell tests and all studies used deionized water (DI).

### 2.2. Synthesis of electrocatalysts

#### 2.2.1. Graphene oxide (GO) synthesis

According to the modified Hummer's method, graphite flakes can be oxidized to produce GO sheets.^50,51^ Typically, graphite (5 g) was added to the concentrated sulfuric acid (98%, 115 ml) into a 1 liter beaker under stirring at −10 °C using an ice bath for 30 minutes. Then, 5 g of sodium nitrate was mixed with the solution and kept under vigorous stirring until homogenous suspension. Subsequently, KMnO_4_ (30 g) was gradually added to the previous mixture for 4 h keeping the temperature of the reaction below 20 °C. After that, the suspension was kept under stirring until the solution turned into a dark green paste. After the elimination of the ice bath, 350 ml of hot water was gently transferred to the paste which caused an increase in temperature to 95 °C. After stirring for 1 h, the solution was completed with cold DI water to 1000 ml and the colour was observed to be brown. Subsequently, the hydrogen peroxide H_2_O_2_ (30 ml) was dropped into the solution for 10 minutes till the formation of yellow colour due to the removal of the residual permanganate and manganese dioxide. To eliminate the metal ions, 1 ml of concentrated HCl solution was stirred with the product for 10 minutes. The produced GO was then centrifuged, thoroughly cleaned with DI water to remove any remaining contaminants, and then dried overnight at 60 °C before being stored for further synthesis and electrochemical experiments.

#### 2.2.2. Electrodeposition of NiCuS/Ni-Foam

Firstly, the Ni-foam was cut into (1 × 1 cm^2^) followed by sonication for 10 minutes in 10 ml of HCl (3 M) to eliminate the surface nickel oxide. Subsequently, the foam was sonicated in acetone for 10 minutes followed by ethanol and deionized water (DI) to eliminate the excess HCl. After 10 minutes of washing with each solvent, Ni-foam pieces were vacuum-dried overnight at 60 °C and weighed before the electrodeposition process (*w*_1_). Consequently, the Ni_*x*_Cu_*y*_S_*z*_ (where, *x* = 0.5, *y* = 0.5, and *z* = 1) was formed by multi-thin layers on the cleaned Ni-foam through the electrodeposition process. The deposition bath consisted of 30 ml of 7 mM of NiCl_2_·6H_2_O, 7 mM of CuCl_2_, and 0.75 M of thiourea was prepared as the electrodeposition electrolyte. A standard three-electrode configuration cell was utilized for the electrodeposition process which consists of a counter electrode, CE (platinum electrode), and the working electrode, WE (NF) *versus* Ag/AgCl reference electrode, RE which was dipped in the deposition bath. The deposition was performed under continuous stirring by applying the cyclic voltammetry from −1.2 to +0.2 V for 5 cycles at a 5 mV s^−1^ scan rate. After this process, the sample was cleaned twice with ethanol before being vacuum-dried at 60 °C for an overnight period. Typically, the electrodeposition of NiS and CuS catalysts was prepared by the same procedure using 30 ml of 14 mM of NiCl_2_·6H_2_O and CuCl_2_, respectively, and 0.75 M of thiourea. Finally, after the electrodeposition process for each material, the loaded NF was washed with water, dried under a vacuum at 60 °C overnight, and weighed (*w*_2_). The loading of active materials was determined from the difference between the loaded NF and the pure one (*w*_2_ − *w*_1_).

The electrodeposition of the metal sulfides occurred as follows:^52^

1 2H_2_O + 2e → H_2_ + 2OH^−^

2 2OH^−^ + SC(NH_2_)_2_ → S^2−^ + OC(NH_2_)_2_ + H_2_O

3 Ni^2+^+ Cu^2+^ + S^2−^ → Ni_*x*_Cu_*y*_S_*z*_

#### 2.2.3. Electrodeposition of Ni_0.5_Cu_0.5_S/rGO on nickel foam

To enable the adsorption of sulfide on the GO surface, a certain amount (50 mg) was sonicated in 30 ml of 0.75 M thiourea solution for 30 minutes. Thereafter, the equivalent masses of NiCl_2_·6H_2_O (7 mM) and CuCl_2_ (7 mM) were added to the GO and thiourea mixture forming the electrodeposition electrolyte. Similarly, the deposition of Ni_0.5_Cu_0.5_S/rGO was utilized by applying the 5 cycles of CV at a scan rate of 5 mV s^−1^ through a voltage window of (−1.2 to +0.2 V). Moreover, the electrodeposition process of Ni_0.5_Cu_0.5_S/50rGO was applied at a scan rate of 5 mV s^−1^ for cycle numbers of 5 and 20 cycles. Besides, the Ni_0.5_Cu_0.5_S/rGO with different masses (5 and 50 mg) were prepared and labeled as Ni_0.5_Cu_0.5_S/5rGO, and Ni_0.5_Cu_0.5_S/50rGO, respectively.

### 2.3. Materials characterization

To reveal the crystal structure of the materials, an X-ray diffraction (XRD) investigation was conducted by the Bruker equipment with monochromated Cu-K_α_ radiation with *λ* of 1.54 and a scan rate from 5 to 70°. A scanning electron microscope (SEM) analysis was used with the Jeol JSM-6510 to confirm the shape and morphology of the fabricated electrodes. X-ray photoelectron spectroscopy (XPS) with a monochromatic (Al-Kα) X-ray source and Energy Dispersive X-ray apparatus (EDX model: INCA Energy 350) were used to analyze the chemical and elemental composition of the manufactured materials, respectively. Using Fourier transform infrared spectroscopy, the chemical compounds or functional groups were identified (FT-IR). A *versastat* potentiostat/galvanostat was used for the electrochemical measurements with a three-electrodes configuration.

### 2.4. Electrochemical tests of supercapacitors

#### 2.4.1. The electrochemical tests of electrodes

Through a potentiostat, the deposited electrodes were tested electrochemically utilizing cyclic voltammetry (CV), galvanostatic charge–discharge (GCD), and electrochemical impedance spectroscopy (EIS) methods. With a platinum counter electrode, the electrochemical characteristics were tested *versus* Ag/AgCl in 1 M KOH. The fabricated electrodes were activated by 50 cycles of the CV technique from 0 to 0.5 V at a 50 mV s^−1^ scan rate before taking the data due to unstable capacitive performance. Afterwards, the CV measurements were analyzed at different scan rates (5, 10, 20, 40, and 50 mV s^−1^) with a potential window from 0 V to 0.5 V followed by the GCD measurements with the same voltage window for various current densities (1–20) A g^−1^. Finally, the EIS measurements were carried out at frequencies ranging from 0.1 to 10^5^ Hz using the open circuit potential (OCP) with an amplitude of 5 mV. To conduct the specific capacity (*C*_s_, C g^−1^) of different as-synthesized electrodes, the CV data at various scan rates were used as follows:^53–58^4
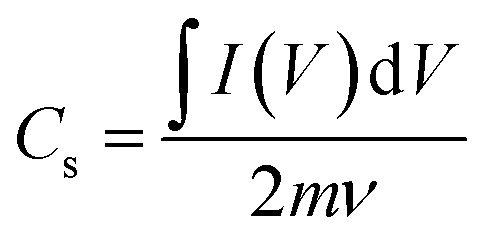
where 
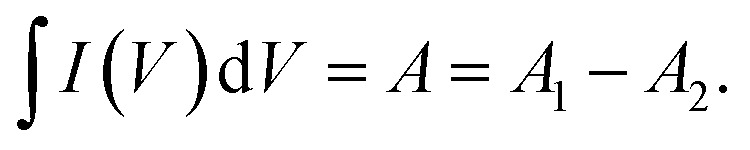
*A* is an integrated area under the CV curve. *A*_1_ is the area under the curve when the supercapacitor is charging while *A*_2_ is the area under the curve when the supercapacitor is discharging. *m* and *ν* are the weight of electrodeposited material on the NF (g), and the applied scan rate (V s^−1^), respectively.

Moreover, the specific capacity (*C*_s_, C g^−1^) and specific capacitance (*C*, F g^−1^) depending on the GCD results can be conducted as follows:^57,59^5
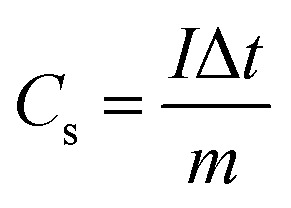

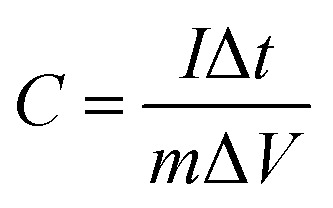
where *I* (A), Δ*t* (s), and *m* (g) are the current density, discharging time, and the mass of deposited active material.

The Nyquist graphs were evaluated as below:^60^6
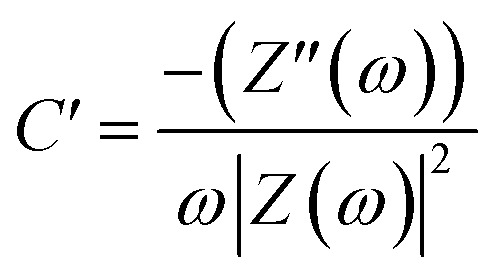
7
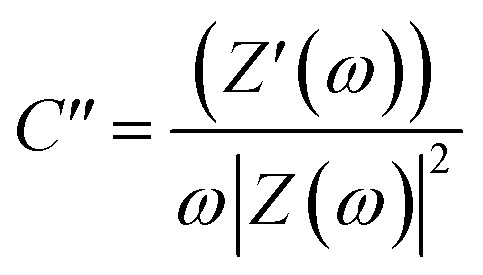
where *ω* = 2π*f* and *f* denotes the frequency in Hz, *Z*(*ω*), *Z*′(*ω*), and *Z*′′(*ω*) are the complex, real, and imaginary impedances, respectively, and *C*′ and *C*′′ are the real and imaginary capacitances.

#### 2.4.2. Electrochemical tests of the supercapacitor cell (Ni_0.5_Cu_0.5_S/50rGO//AC)

Firstly, a certain amount of activated carbon and carbon black were mixed with PVDF with a weight ratio of 8 : 1 : 1, respectively, and then ultrasonicated in 50 μl of *N*-methyl-2-pyrrolidone (NMP) as a solvent till the formation of a slurry. A 1 × 1 cm^2^ piece of cleaned Ni-foam was covered by a thin layer of the slurry, vacuum-dried at 60 °C overnight, and used as a cathode for the asymmetric supercapacitor cell (ASC). The ASC was fabricated using a piece of filter paper as a separator, an activated carbon negative electrode, a NiCuS/50rGO positive electrode, and a 1 M KOH electrolyte. To keep the charge balance between the cathode and anode, the weight ratios of the anode (NiCuS/50rGO) and cathode (active carbon) were modified using the equation below:

8 *Q*^+^ = *Q*^−^

9 *Q* = *m* × Δ*V* × *C*

10
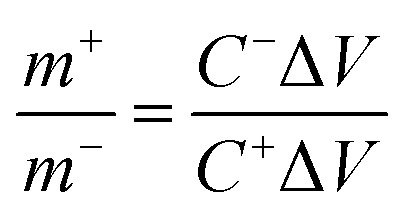
where *C* (F g^−1^), *V* (V), *Q*^+^, and *Q*^−^, respectively, are the specific capacitance, voltage window throughout the GCD technique, positive charge, and negative charge derived from the three-electrode configuration. Additionally, it is possible to further transform the electrode's specific capacity into specific capacitance (F g^−1^) when employing exclusively capacitive materials (AC) in devices.^61^ Furthermore, m (g) is the loading mass of the supercapacitor device's components, which was calculated from the total of the masses loaded on the NF surface for the cathode and anode (3.06 mg cm^−2^).

Finally, the cell power and energy densities were conducted as follows:11
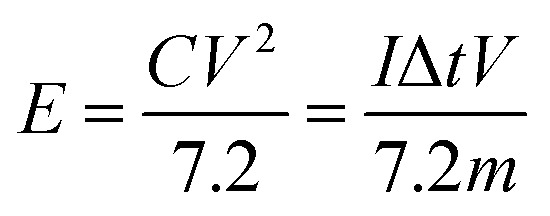
12
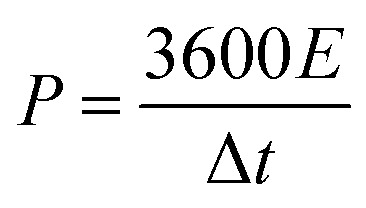
where *P* (W kg^−1^), *E* (W h kg^−1^), *V* (V), *C* (F g^−1^), Δ*t* (s), and *m* (g) are the power density, energy density, voltage window, specific capacitance, time of discharge, and the combined mass of negative and positive electrodes.

## 3. Results and discussions

### 3.1. Structural characterization

**Fig. 1:**
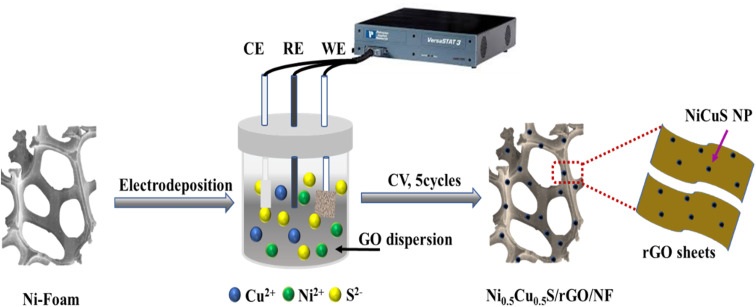
Scheme of the electrodeposition performance of Ni_0.5_Cu_0.5_S/rGO on NF surface.

The simple electrodeposition method was used to prepare the nanosheet structure of Ni_0.5_Cu_0.5_S/rGO/NF electrode from CuCl_2_, NiCl_2_·6H_2_O, (NH_2_)_2_CS, and the as-synthesized GO as precursors (Fig. 1). The deposition process on the highly conductive porous NF substrate contains three procedures; firstly, the as-synthesized GO with various amounts was dispersed in thiourea solution to allow the adsorption of sulfide on the surface of GO and used as electrodeposition electrolyte. Secondly, the cyclic voltammetry allows the GO in solution to be reduced and deposited onto the NF surface. Thirdly, the metal precursors were dissolved in the electrolyte and the cations went through the diffusion layer by driving force of the applied potential and reacted with sulfide ions to be deposited onto the rGO/NF. A uniform coating of active materials over the NF surface was achieved by the CV technique which was applied with limiting sweeping cycles (5 cycles) to control the thickness of the deposited materials. It demonstrates that the very porous electrode is created by growing the Ni_0.5_Cu_0.5_S interconnected rGO nanosheet arrays directly on the conductive NF. Moreover, the thickness of the deposited active materials can be estimated from the electrochemistry measurements using Faraday–Coulombs' law as follows;^62,63^13
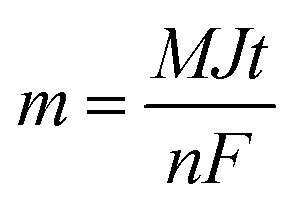
where *m* is the weight of the deposited active materials, *M* and *ρ* are the molecular weight and density of the deposited material, *J* is the current in amperes flow through the solution, *n* is the number of electrons in the electrochemical reaction, *t* is the time of current flow through the solution during the deposition, and *F* is Faraday's constant, equal to 96 500 C (or 96 500 A s). The obtained deposited material was divided by the density of the material and the area of the electrode exposed to the ionic bath (1 cm × 1 cm of NF). Meanwhile, the thickness of the deposited active materials was estimated for the electrodeposition at 5 and 20 CV cycle numbers. The ratio between the thickness after 20 cycles and that after 5 cycles was found to be 5.5 (91.095 μm for 20 cycles and 15.7 μm for 5 cycles).

**Fig. 2:**
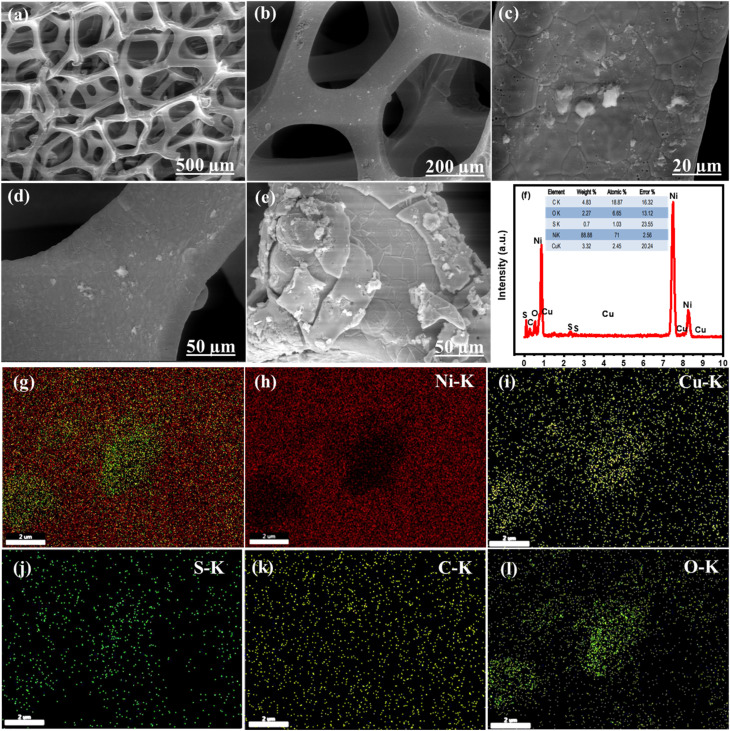
SEM images of (a) pristine NF, (b) Low magnification SEM images and (c and d) high magnification SEM images of Ni_0.5_Cu_0.5_S/50rGO/NF deposited at 5 CV cycles, (e) high magnification SEM images of Ni_0.5_Cu_0.5_S/50rGO/NF deposited at 20 CV cycles, (f) EDX spectrum of Ni_0.5_Cu_0.5_S/50rGO/NF deposited at 5 CV cycles, and the EDX mapping of (g) Ni_0.5_Cu_0.5_S/50rGO/NF deposited at 5 CV cycles, (h) Ni, (i) Cu, (j) S, (k) C, and (l) O.

The growth of the materials on the NF surface through the electrodeposition process was confirmed using different characterizations. Finally, the morphology and microstructure of the NF and Ni_0.5_Cu_0.5_S/rGO/NF were studied by SEM imaging. The SEM image of the bare nickel NF is displayed in Fig. 2a which shows a 3D cross-linked porous structure with a smooth surface that can provide a significant specific surface area per unit electrode area for coating rGO structures.^64^ The nickel foam structure with wrinkled graphene topography coating and Ni_0.5_C_0.5_S distribution is displayed in Fig. 2b–e. The low SEM image (Fig. 2b) and high magnification images (Fig. 2c and d) of deposited Ni_0.5_Cu_0.5_S/50rGO at controllable CV cycles (5 cycles) show that graphene is grown on the NF with a uniform and small thickness. The nickel foam skeleton may give a higher porosity and bigger surface area compared to the traditional graphene sheet, thereby preventing the aggregation of graphene nanosheets.^65^ Compared to Fig. 2d, the high magnification image of Ni_0.5_Cu_0.5_S/50rGO at 20 CV cycles (Fig. 2e) shows the high thickness and aggregation of rGO on the NF surface. The precise morphologies of the nickel foam skeleton covered with Ni_0.5_Cu_0.5_S/5rGO nanosheet deposited at 5 CV cycles are shown in Fig. S1,† demonstrating that GO solution can easily penetrate the nickel foam's micropores and adhere well to its surface.^65^ Moreover, the images display the successful deposition of the Ni_0.5_Cu_0.5_S nanoparticles with a small size on the rGO-coated NF surface. The EDX analysis (Fig. 2f) was conducted to demonstrate the elemental composition of the Ni_0.5_Cu_0.5_S/rGO/NF electrode which showed that Ni was highly concentrated there since the substrate was made of Ni-foam. Besides, the observed C, O, Ni, Cu, and S elements from EDX indicate the successful synthesis of Ni_0.5_Cu_0.5_S/rGO/NF. Meanwhile, the EDX mappings can confirm that these components are dispersed evenly across the substrate (Fig. 2g–l). The coexistence of these components in the electrodeposited materials without any additional peaks of foreign elements indicates their high purity. Based on these results, the electrodeposition with low cycles (5 cycles) was preferred to obtain the low thickness of the deposited materials on the NF surface.

**Fig. 3:**
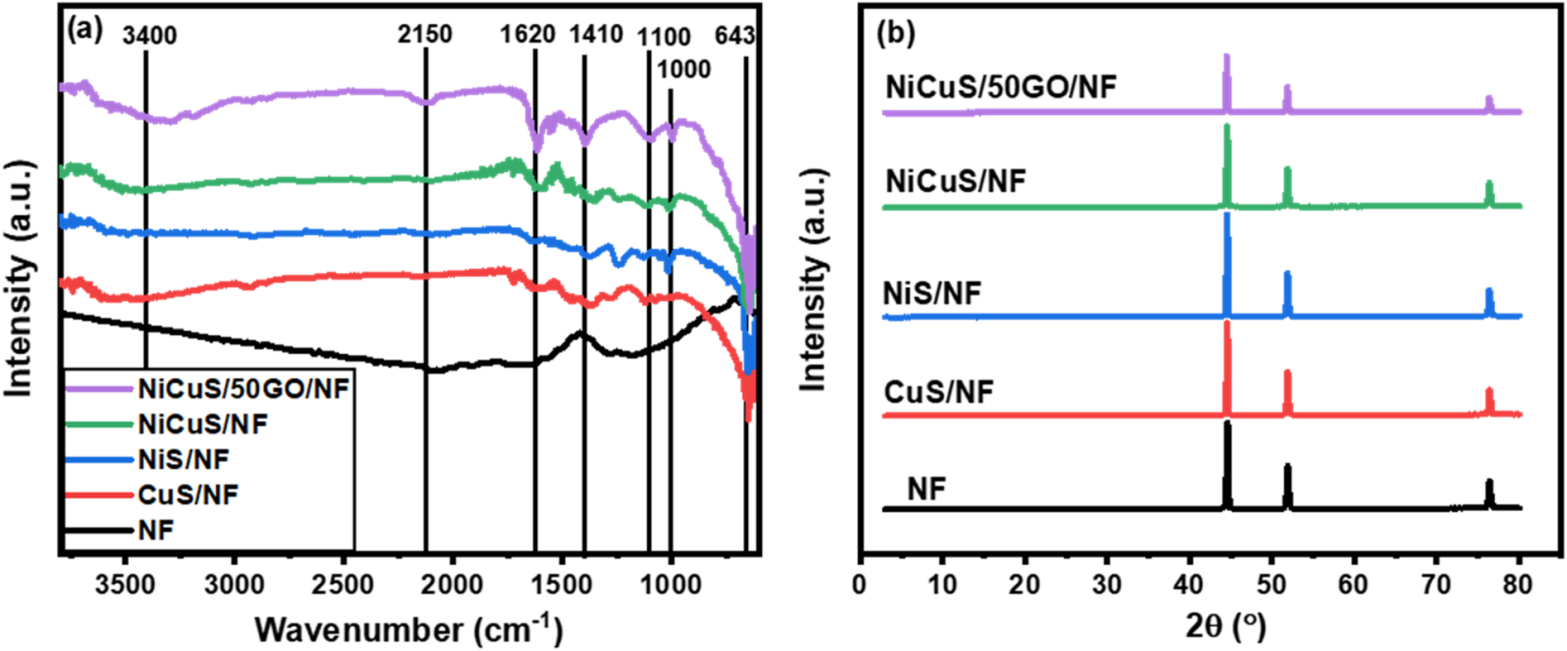
(a) XRD pattern and (b) high-magnification XRD pattern of NF, CuS/NF, NiS/NF, Ni_0.5_Cu_0.5_S/NF, and NiCuS/rGO/NF.

The evaluation of functional groups in all electrodeposited materials on NF was performed using the FT-IR spectra which are depicted in Fig. 3a. The figure shows that there are no apparent vibrational bands in the spectrum of bare nickel foam indicating that the foam was completely free from the organic groups after the washing process.^66^ The characteristic peak at 1000 cm^−1^ in the CuS/NF spectrum assigns the H–O–H bending indicating the absorbed water molecules in the product, whereas the characteristic peak Cu–S stretching modes appeared at 638 cm^−1^.^67^ Due to atmospheric carbon and oxygen being absorbed, the peak of the C–O stretching mode appeared at 1100 cm^−1^.^68^ In the NiS/NF spectrum, the peak around 640 cm^−1^ is due to the stretching vibration mode of Ni–S, and the other vibration bands appeared around 1000, 1100, 1278, and 1620 cm^−1^ are characteristic of the bending mode of OH, stretching mode of C–O, bending mode of C–H and stretching mode of OH, respectively.^69^ For Ni_0.5_Cu_0.5_S/NF, the co-existence of the above-all peaks of CuS and NiS in the ternary system confirmed the successful construction of the NiCuS electrode. Moreover, the spectrum of Ni_0.5_Cu_0.5_S/rGO/NF displays the main peaks of GO at 3400 cm^−1^ corresponding to free OH, while the peaks at 1086, 1410, 1620, and 2150 cm^−1^ are evidence of the existence of the C–O, C=C, C=O, and C–H functional groups, respectively.^50^ Besides, the low intensity of these peaks confirms the reduction of GO through the electrodeposition process.^70^

To further clarify the phase structure of the bare nickel foam as well as the electrodeposited materials, XRD experiments were applied and displayed in Fig. 3b. The figure exhibits the characteristic XRD peaks of bare nickel foam substrate at 2*θ* of 44.49°, 51.87°, and 76.45° which are, respectively, indexed to the (111), (200), and (220) Miller indices.^71^ For the XRD patterns of electrodeposited materials, the low intensity of the XRD peaks of NF is due to the electrodeposition process. Moreover, the characteristic peaks corresponding to CuS, NiS, Ni_0.5_Cu_0.5_S, or Ni_0.5_Cu_0.5_S/rGO were not clearly observed, which is possibly ascribed to the lower crystallinity due to the little loading amount of electrodeposited active materials on the NF surface indicating the small thickness and size of these materials.^72,73^ However, high-magnification XRD patterns (Fig. S2†) show slightly lower 2*θ* angles for electrodeposited materials in comparison to the main peaks of nickel foam. For CuS/NF, the figure shows the characteristic peaks of CuS at around 2*θ* of 27.49, 29.10, 31.25, 32.91, 40.44, and 59.48° which are indexed to (101), (102), (103), (006), (110), and (108) planes, respectively.^74^ Moreover, the NiS/NF pattern displays the major peaks of the NiS phase at 2*θ* of 27.1, 30.4, 32.3, 35.3, 36.8, and 54.7° which is consistent with the JCPDS 012-0041.^75^ All of the peaks associated with NiS and CuS in the Ni_0.5_Cu_0.5_S/NF pattern are slightly different from their corresponding peaks found in pure materials, suggesting a strong attraction between NiS and CuS particles to create the Ni_0.5_Cu_0.5_S composite.^76^ Meanwhile, the presence of any other peaks in all prepared electrodes may be due to the oxidation of the nickel foam surface to form NiO.^76^ Finally, the XRD pattern of Ni_0.5_Cu_0.5_S/rGO/NF exhibits the broad peak of the rGO at 2*θ* of 25.8° (002) suggesting the GO reduction through the electrodeposition process.^51,77^ Thus, XRD studies reveal the successful deposition of metal sulfides on NF surface, reduction of GO, and fabrication of metal sulfide and rGO nanocomposites which agrees with the FT-IR study.

**Fig. 4:**
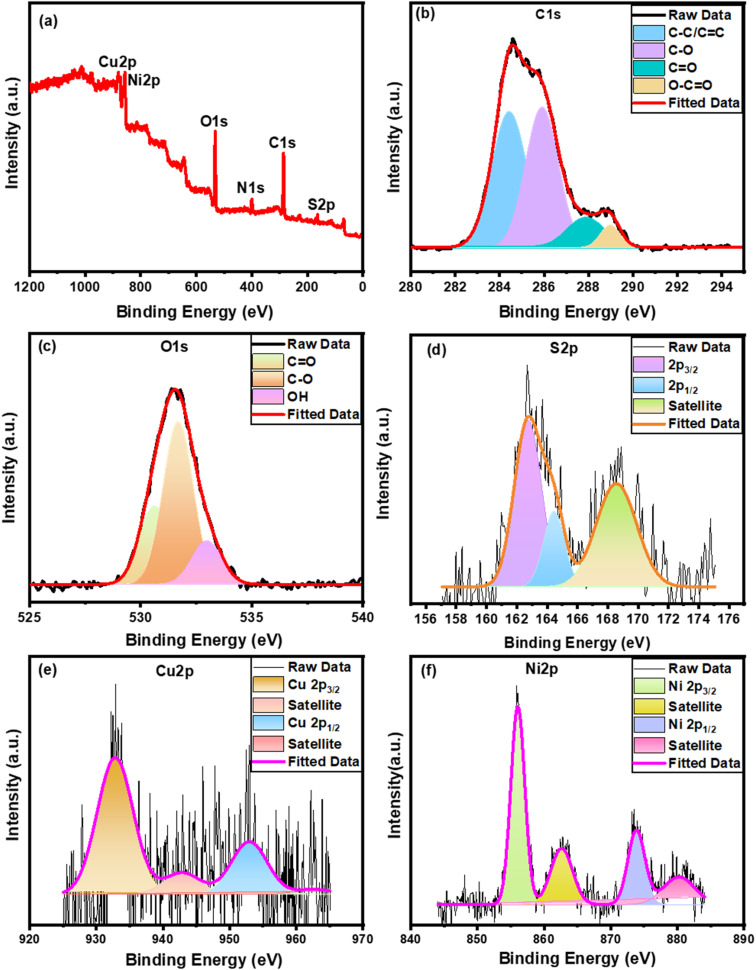
XPS spectra of the Ni_0.5_Cu_0.5_S/rGO/NF; (a) total survey, high resolution of (b) C 1s, (c) O 1s, (d) S 2p, (e) Cu 2p, and (f) Ni 2p.

Furthermore, the XPS approach (Fig. 4a) reveals the presence of C 1s, O 1s, S 2p, Cu 2p, and Ni 2p on the Ni substrate surface. The high-resolution spectrum of C 1s can be fitted into four peaks (Fig. 4b) at 288.98 eV (O–C=O), 287.89 eV (C=O), 285.96 eV (C–O), and 284.49 eV (C=C/C–C).^50,78^ Besides, Fig. 4c reveals the O 1s spectrum which consists of three main peaks at a binding energy of 532.98, 531.64, and 530.68 eV for OH, C–O, and C=O, respectively.^50,79^ Furthermore, the S 2p spectrum (Fig. 4d) shows one main peak at ∼163 eV and a satellite peak at ∼168.58 eV while the overlapped main peak was fitted into two Gaussian peaks: S 2p_3/2_ (at 162.5 eV) and S 2p_1/2_ (at 164.4 eV) confirming the bonding of sulfur with metals (Cu–S and Ni–S bonds) and the presence of low coordination numbers of sulfur ions, respectively.^71^ Two satellite peaks for the Cu 2p (Fig. 4e) were evident at 943.1 and 963.3 eV in the core spectra, while two peaks for the Cu 2p_1/2_ and Cu 2p_3/2_ were discernible at 953.8 and 932.9 eV, respectively indicating the divalent state of Cu.^80^ On the other hand, the two spin–orbit coupling peaks at 873.8 and 855.9 eV belonging to Ni 2p_1/2_ and Ni 2p_3/2_, respectively, appeared with two satellite peaks at 862.6 and 880.3 eV which clearly indicate the existence of Ni in the divalent state (Fig. 4f).^80^

### 3.2. Electrochemical performance

**Fig. 5:**
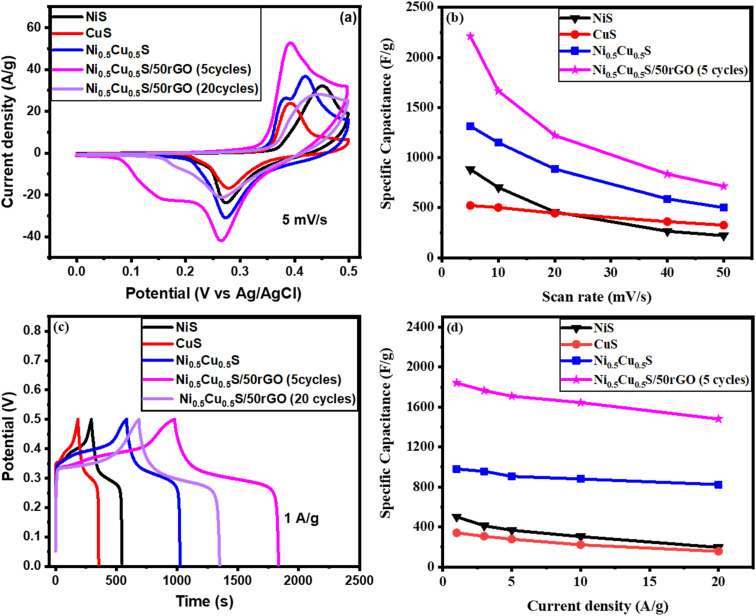
(a) CVs and (c) GCD of all deposited electrodes, and the specific capacity (*C*_s_) at (b) various scan rates, and (d) various current densities.

The Ni_0.5_Cu_0.5_S/rGO that was deposited on the NF surface was electrochemically tested and compared to NiS, CuS, and Ni_0.5_Cu_0.5_S that had been produced in 1 M KOH under the same conditions using a 3-electrode system, as seen in Fig. 5. Firstly, Fig. 5a compares the results of the cyclic voltammetry (CV) tests performed on the manufactured catalysts using an operating potential window of 0–0.5 V with a scan rate of 5 mV s^−1^. The figure shows one pair of redox peaks for each curve, suggesting the involvement of battery-type components in the redox reaction. The Ni_0.5_Cu_0.5_S/50rGO electrode shows redox peaks at ∼0.39 V (oxidation) and 0.26 V (reduction) indicating the redox process. The figure shows one pair of redox peaks for each curve due to the reaction of the alkaline electrolyte with the active materials of the electrode as follows:^81–83^NiS + OH^−^ → NiS–OH + e^−^CuS + OH^−^ ↔ CuS–OH + e^−^NiCuS + 2OH^−^ ↔ NiS–OH + CuS–OH + 2e^−^CuS–OH + 2OH^−^ ↔ CuSO + H_2_O + e^−^NiS–OH + 2OH^−^ ↔ NiSO + H_2_O + e^−^

At a 5 mV s^−1^ scan rate, the NiS catalyst shows one oxidation peak at 0.43 V and CuS shows one oxidation peak at 0.37 V while Ni_0.5_Cu_0.5_S shows two oxidation peaks that were typical of secondary sulfides. It was noticed that after the incorporation of NiS and CuS, the redox peaks of the NiS electrode moved to a more negative position. The peak with a greater current may be attributed to the NiS, while the peak with a smaller current may be attributed to the CuS.^71^ Moreover, the CV curve of each electrode shows distinct redox peaks concerning the redox reactions indicating the (battery type) behaviour of the three electrodes. On the other hand, Ni_0.5_Cu_0.5_S/50rGO (5 cycles) has the largest CV area among all electrodes, suggesting that the addition of rGO has enhanced the Ni_0.5_Cu_0.5_S performance. The integrated area of Ni_0.5_Cu_0.5_S/50rGO CV was greater than that of all deposited electrodes indicating the high charge storage and capacitance. The figure also shows that two overlapping peaks of Ni_0.5_Cu_0.5_S appear in Ni_0.5_Cu_0.5_S/50rGO as a broad peak with the appearance of a double-layer portion while the two peaks were still present in Ni_0.5_Cu_0.5_S/5rGO (5 cycles) due to the low amount of rGO (Fig. S3†). Compared to the CV curve of the Ni_0.5_Cu_0.5_S/50rGO (5 cycles) electrode, Ni_0.5_Cu_0.5_S/50rGO (20 cycles) has a different shape with higher oxidation potential and low current density indicating the lower charge storage due to the aggregation of rGO on the NF surface (Fig. 5a and S3†). No difference of reduction peaks between Ni_0.5_Cu_0.5_S/5rGO and Ni_0.5_Cu_0.5_S/50rGO (5 cycles) is clear but compared to the reduction peak of Ni_0.5_Cu_0.5_S/50rGO (20 cycle) shifted to a negative position. The deposition at low CV cycles enables the thin layer formation which provides the easier penetration of electrolytes to facilitate electron and charge transfer.^81^ Moreover, the cyclic voltammetry studies of NiS, CuS, Ni_0.5_Cu_0.5_S, Ni_0.5_Cu_0.5_S/5rGO, Ni_0.5_Cu_0.5_S/50rGO (5 cycles) and Ni_0.5_Cu_0.5_S/50rGO (20 cycles) electrodes were performed at different scan rates of 5–50 mV s^−1^ with an operating voltage window of (0–0.5 V). Fig. S4a–f† shows that the reduction and oxidation peaks for all electrodes shifted to lower and higher potentials, respectively, with the increase of scan rate in regard to the internal resistance of the electrode and the quick interfacial rate kinetics demonstrating that the redox processes are diffusion-controlled. Meanwhile, at low scan rates, Ni_0.5_Cu_0.5_S (Fig. S4c†) and Ni_0.5_Cu_0.5_S/5rGO (Fig. S4d†) maintained the two peaks of Ni_0.5_Cu_0.5_S while the two peaks overlapped at the high scan rates. Moreover, Fig. S4e† shows that the current increased with the increase of the rGO amount in Ni_0.5_Cu_0.5_S/50rGO (5 cycles) electrode, while the current of Ni_0.5_Cu_0.5_S/50rGO (20 cycles) decreased due to the high thickness of rGO in NF. Besides, Fig. 5b displays the effect of the scan rate on the specific capacitance values for the tested electrodes which shows that at all scan rates, the Ni_0.5_Cu_0.5_S/50rGO (5 cycles) exhibited a greater specific capacitance than other electrodes. The specific capacitance of NiS, CuS, Ni_0.5_Cu_0.5_S, and Ni_0.5_Cu_0.5_S/50rGO (5 cycles) at a scan rate of 5 mV s^−1^ reached 882.05, 521, 1314.39, and 2211.029 F g^−1^ (441.02, 260.7, 657.2, and 1105.5 C g^−1^), respectively. The synergistic interaction between rGO and the bimetallic sulfides may account for the exceptionally high specific capacity of the Ni_0.5_Cu_0.5_S/50rGO.^71^ Also, Fig. 5b exhibits that the increase in scan rate leads to a decrease in the specific capacity of all electrodes, however, the Ni_0.5_Cu_0.5_S/rGO electrode maintained its greater capacity of 908 F g^−1^ even at a high scan rate. The specific capacity that was obtained, as shown in Table S1,† is higher than those that were published in the literature for the different carbon-based materials and metal sulfides. From the EDL capacitance, which was obtained from the slope of the current-scan rate curve, the electrochemically active surface area (ECSA) can be determined (Fig. S5†) and was found to be 8.33, 38.4, 77.66, and 136 cm^2^ g^−1^ for NiS, CuS, Ni_0.5_Cu_0.5_S, Ni_0.5_Cu_0.5_S/50rGO (5 cycles), respectively. The results give evidence that Ni_0.5_Cu_0.5_S/50rGO electrode has the largest ECSA compared to other electrodes due to the existence of rGO.

GCD measurements were carried out for all deposited materials since the charge and discharge properties are the primary indicators of good charge storage and transfer in the working electrodes. With a potential window (0–0.5), Fig. 5c explains the GCD measurements of all fabricated electrodes at 1 A g^−1^ which are identical to the GCD curves of a typical pseudocapacitance performance. Compared to all fabricated electrodes, Ni_0.5_Cu_0.5_S/50rGO (5 cycles) electrodes showed longer discharge times and more symmetric forms indicating larger high-energy storage capacitors and electrochemical reversibility owing to the low resistance of the charge-transfer and the fast electron mobility and collection which attributed to the addition of conductive rGO.^84^ The correlation between the redox peaks and voltage plateaus of the CV curves so far demonstrates the characteristic coexistence of EDL-faradaic (redox) nature. Moreover, Fig. S6† displays a comparison between the GCD curves of the Ni_0.5_Cu_0.5_S/5rGO (5 cycles), Ni_0.5_Cu_0.5_S/50rGO (5 cycles), and Ni_0.5_Cu_0.5_S/50rGO (20 cycles) which confirms the high electrical performance of the Ni_0.5_Cu_0.5_S/50rGO (5 cycles) due to the high content and the small thickness of rGO on the NF surface. The GCD measurements were tested for all electrodes at various current densities (1–20 A g^−1^) (Fig. S7a–f†) and it was noticed that the high current density caused a remarkable drop in discharge times for all electrodes. The GCD curves are very symmetric at the different current densities owing to the highly reversible redox reactions of the deposited electrodes on the charge/discharge process, suggesting great coulombic efficiency. Concurrently, the GCD curves at various current densities were used to conduct the specific capacity of the manufactured electrodes which were found to be ∼502.4, 341.4, 980.4, and 1840.2 F g^−1^ (251.2, 170.7, 490.2, and 920 C g^−1^) for NiS, CuS, Ni_0.5_Cu_0.5_S, and Ni_0.5_Cu_0.5_S/50rGO, respectively (Fig. 5d). The high specific capacity was observed for the Ni_0.5_Cu_0.5_S/50rGO electrode by the combination of Cu and Ni sulfides and the diffusion of Ni–Cu–S through graphene and NF layers which increased the charge transfer and electrical conductivity. Note that further electrochemical kinetics studies were performed for the materials deposited by 5 cycles of the CV technique depending on the CV and GCD results.

**Fig. 6:**
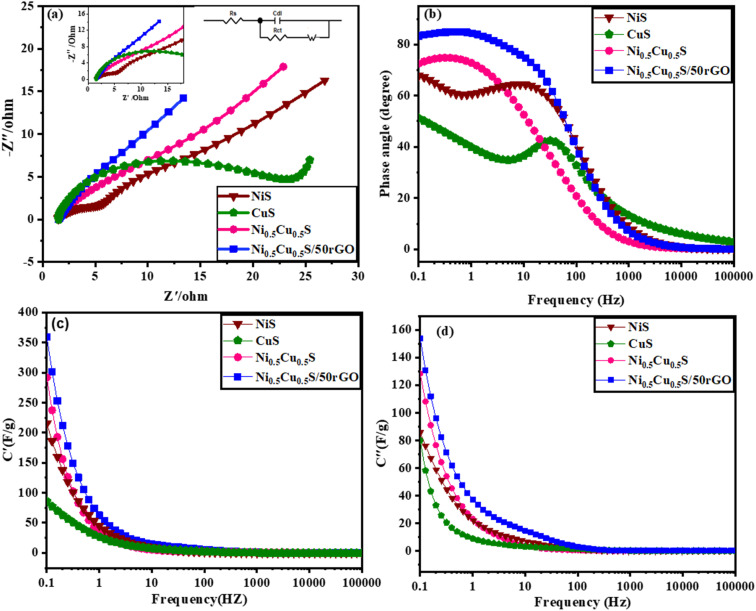
Nyquist plots of the fabricated electrode (the insets are the semicircle of materials) (a), Bode plots (b), and the real (c) and imaginary (d) capacitances of electrocatalysts *versus* frequency.

Electrochemical impedance spectroscopy (EIS) was conducted to clarify the internal mechanism of the electrochemical properties of the interfaces between the electrolyte and electrode surface. The Nyquist plot from 0.1 Hz to 10^5^ Hz was obtained from the fitting of EIS measurements by the equivalent circuit model (Randles circuit) (Fig. 6a). It was noticed that each Nyquist plot has two components: a semicircle in the high-frequency region and a straight line in the low-frequency region.^85^ Meanwhile, the equivalent series resistance (*R*_s_) of the electrode–electrolyte, which is composed of multiple resistances (*e.g.* the ionic resistance of the substrate, electroactive material, and electrolyte interface) was estimated from the left intercept of the semi-circle region.^86^ The charge transfer resistance (*R*_ct_) is conducted from the diameters of semi-circles for analyzing the faradaic kinetics.^87^ The ion diffusion resistance (*R*_w_) is represented by the straight line slope that depicts the transfer and diffusion kinetics of electrolytes in the electrodes. Fig. 6a shows that the pure Ni_0.5_Cu_0.5_S electrode exhibited an *R*_s_ value of 1.36 Ω and *R*_ct_ value of 14.56 Ω while the Ni_0.5_Cu_0.5_S/50rGO electrode exhibited 1.6 and 4.18 Ω, respectively. This suggested that the addition of rGO to Ni_0.5_Cu_0.5_S electrodes increases their electrical conductivity while also providing rapid charge transfer, which encourages the use of active compounds and hence quick electrochemical kinetics. Furthermore, the diffusion kinetics were studied from Bode graphs (Fig. 6b) which show the phase angle values of the NiS (54°), CuS (48°), Ni_0.5_Cu_0.5_S (63°), and Ni_0.5_Cu_0.5_S/50rGO (73°) electrodes at a low frequency of 10^5^ Hz. The rate-limiting behaviour may be studied by looking at variations, where phase angle (45°) for a diffusion-limited response, and (90°) for a completely capacitive response.^88^ The angle of NiS, CuS, and Ni_0.5_Cu_0.5_S is closer to 45 which indicates the diffusion-controlled mechanism is more dominant while with the addition of rGO, the angle of Ni_0.5_Cu_0.5_S/50rGO increased to (73°) which is closer to the ideal capacitor value (90°) confirming the surface capacitive properties of Ni_0.5_Cu_0.5_S/50rGO nanocomposites once again. So, Bode's plot is very agreeing with Dunn's approach and the value *b* in the power law. Additionally, the discharging and charging rates of electrodes are frequently analyzed using the relaxation time constants *τ*_0_ that can be calculated as follows; *τ*_0_ = ½π*f*_0_ (where *f*_0_ is the frequency at 45°) and were found to be 4, 691, 14, and 2.58 ms, for NiS, CuS, Ni_0.5_Cu_0.5_S, and Ni_0.5_Cu_0.5_S/50rGO, respectively. The smaller “*τ*_0_” values of Ni_0.5_Cu_0.5_S/50rGO electrodes showed rapid ion diffusion and transport kinetics and thus enhanced the electrochemical characteristics of the electrodes. Finally, Fig. 6c and d present the plot of actual capacitance (*C*′) and the virtual capacitance (*C*′′) *versus* the frequency and show that Ni_0.5_Cu_0.5_S/50rGO electrode exhibits superior capacitive performance among all electrodes.

It is commonly known that EDLC and faradaic charge storage methods coexist in a supercapacitor system but depending on the active electrode material and electrolyte applied, one may dominate the other at a given moment. The redox reactions result from the ions incorporation into the layers or channels of the electrode from the electrolyte on the surface together with a faradaic charge transfer without a phase difference in crystals.^89^ EDL process (non-faradaic) is determined by the charge separation in a Helmholtz double layer at the electrodes–electrolyte interface. The high surface area of the electrode and electrolyte interface allows for the production of EDL capacitors with large storage capacities. At low scan rates, the diffusive behaviour is more prominent because ions have enough time to interact with the surface of the electrode while at high scan rates promote the capacitive behaviour.^90^ The ratios of capacitive/diffusion-controlled contributions might be conducted using Dunn's approach depending on the redox current of the CV curve at a certain potential (*i*(*V*)) as follow:^86^

14 *i*(*V*) = *k*_1_*ν* + *k*_2_*ν*^1/2^

This formula can be modified as

15 *i*(*V*)/*ν*^1/2^ = *k*_2_ + *k*_1_*ν*^1/2^

The *i*(*V*)/*ν*^1/2^ against *ν*^1/2^ plots are used to estimate the values of *k*_2_ (intercept) and *k*_1_ (slope). The capacitive current (*i*_c_) is denoted by *k*_1_*ν* whereas the diffusion-controlled current (*i*_d_) is denoted by *k*_2_*ν*^1/2^.

**Fig. 7:**
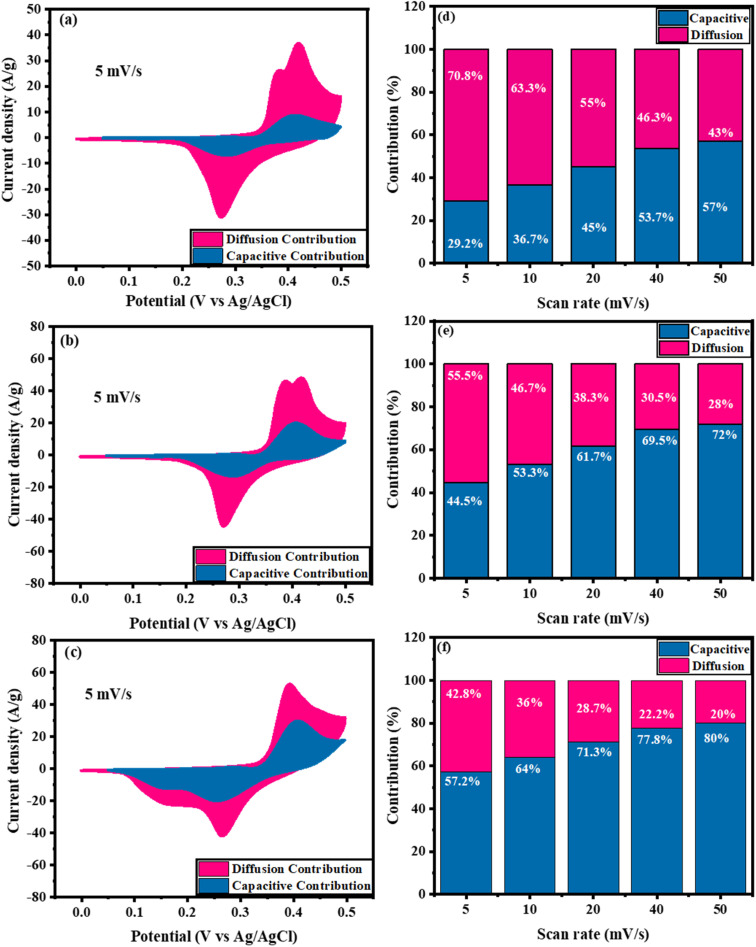
(a–c) CV plots of diffusion/capacitive-controlled contributions at 5 mV s^−1^ and (d–f) the ratio of diffusion/capacitive-controlled contributions as a function of scan rate for Ni_0.5_Cu_0.5_S, Ni_0.5_Cu_0.5_S/5rGO, and Ni_0.5_Cu_0.5_S/50rGO (5 cycles).

Fig. 7a–c displays a comparison between the capacitive and diffusion contributions of Ni_0.5_Cu_0.5_S, Ni_0.5_Cu_0.5_S/5rGO, and Ni_0.5_Cu_0.5_S/50rGO (5 cycles) at 5 mV s^−1^. The ratios of capacitive contributions to diffusion contributions were found to be 29.2% to 70.8% for Ni_0.5_Cu_0.5_S (Fig. 7a), 44.5% to 55.5% for Ni_0.5_Cu_0.5_S/5rGO (Fig. 7b), and 57.2% to 42.8% for Ni_0.5_Cu_0.5_S/50rGO (Fig. 7c) which indicating the diffusion-controlled mechanism of Ni_0.5_Cu_0.5_S and conversely the capacitive-controlled mechanism for Ni_0.5_Cu_0.5_S/rGO composites. Fig. 7d–f, respectively, illustrate the ratios of diffusion/capacitance-controlled contributions of the Ni_0.5_Cu_0.5_S, Ni_0.5_Cu_0.5_S/5rGO, and Ni_0.5_Cu_0.5_S/50rGO as a function of scan rate (5–50 mV s^−1^) which displays that the surface capacitive mechanism dominates for all electrodes at high scan rates. Moreover, Fig. S8a† shows the CV plots of capacitive/diffusion-controlled contributions at 5 mV s^−1^ for Ni_0.5_Cu_0.5_S/50rGO (20 cycles) which were found to be 25% and 75%, respectively. The low value of the capacitive contribution in Ni_0.5_Cu_0.5_S/50rGO (20 cycles), compared to Ni_0.5_Cu_0.5_S/50rGO (5 cycles) was due to the agglomeration of the GO on the NF surface. Fig. S8b† shows the ratio of diffusion/capacitive-controlled contributions as a function of scan rate which displays that the capacitive contributions increase with the increase of the scan rate.

Moreover, the power law relationship determines the energy storage characteristics of the deposited materials.^91–94^

**Fig. 8:**
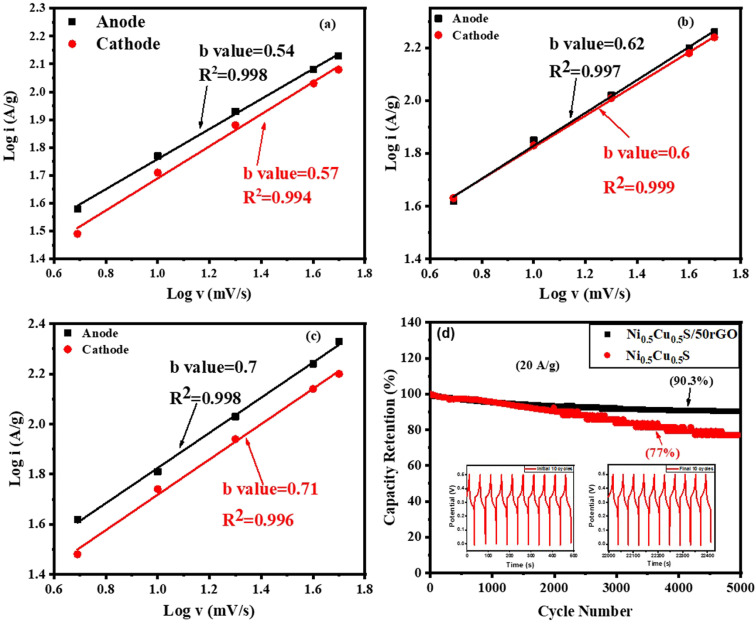
The relationship between the log(*i*) and the log(*ν*) of (a) Ni_0.5_Cu_0.5_S, (b) Ni_0.5_Cu_0.5_S/5rGO, and (c) Ni_0.5_Cu_0.5_S/50rGO and (d) cycling stability comparison of Ni_0.5_Cu_0.5_S and Ni_0.5_Cu_0.5_S/50rGO electrodes at 20 A g^−1^. (The insets are the initial and final ten GCD cycles.)

16 *i* = *av*^*b*^

where “*a*” is a variable that may be changed, and “*b*” is a factor that is highly influenced by the *i*_c_ or *i*_d_ relative contribution. The diffusion and the surface capacitive performances, respectively, are clearly dominant when “*b*” has a value of 0.5 or 1. From the anodic and cathodic peaks of the Ni_0.5_Cu_0.5_S electrode (Fig. 8a), the “*b*” values were conducted and found to be 0.54 and 0.57, respectively, indicating that all sulfide electrodes largely employ a diffusion mechanism for charge storage. While the high “*b*” values of the Ni_0.5_Cu_0.5_S/5rGO and Ni_0.5_Cu_0.5_S/50rGO (5 cycles) electrodes (Fig. 8b and c) which were found to be (0.62 and 0.6) and (0.7 and 0.71), respectively, indicating that the capacitive mechanism of the Ni_0.5_Cu_0.5_S/50rGO electrode for the charge storage due to the incorporation of rGO which agrees well with the above electrochemical tests.^95,96^ The anodic and cathodic peaks of the Ni_0.5_Cu_0.5_S/50rGO (20 cycles) electrode (Fig. S9†) show a low value of “*b*” (0.69) compared to that of Ni_0.5_Cu_0.5_S/50rGO (5 cycles) confirming the Dunn results.

Besides, the long-term cyclic stability of Ni_0.5_Cu_0.5_S/50rGO and pure Ni_0.5_Cu_0.5_S, NiS, and CuS electrodes were studied. Fig. S10† displays the cyclic stability of NiS and CuS electrodes for 5000 cycles at 20 A g^−1^, which exhibited a capacity retention of 64.5% and 56.4% respectively. On the other hand, Fig. S11† demonstrates that after 5000 cycles at 20 A g^−1^, the Ni_0.5_Cu_0.5_S/50rGO electrode still retained 97.3% of the initial capacity, but the pure Ni_0.5_Cu_0.5_S only retained 82% (Fig. 8d). The Ni_0.5_Cu_0.5_S/50rGO electrode GCD curves also keep their unchanged curves after the first and last ten stability cycles (insets in Fig. 8d), indicating strong cyclic stability. Moreover, the figure proved that the weight of rGO added during the synthetic process affects the capacitance retention of electrodes. Additionally, it is higher than the pure Ni_0.5_Cu_0.5_S, NiS, and CuS electrodes, and it competes favourably with those of previously reported sulfide electrodes (Table S1†). The Ni_0.5_Cu_0.5_S/rGO deposited electrode performs more electrochemically due to the following factors: first, graphene sheets with well-distributed Ni_0.5_Cu_0.5_S may be prevented from aggregating together and restacking during the fast charge/discharge process which produces considerably shorter diffusion and migration pathways and thus high specific capacitance. Second, the higher surface area increased the active sites and facilitated the movement of electrolyte ions, improving rate capability and cycle stability. Third, the exact amount of rGO in the electrode increased the electrical conductivity owing to its superior conductivity, mechanical capabilities, and electrochemical stability.^97^

**Fig. 9:**
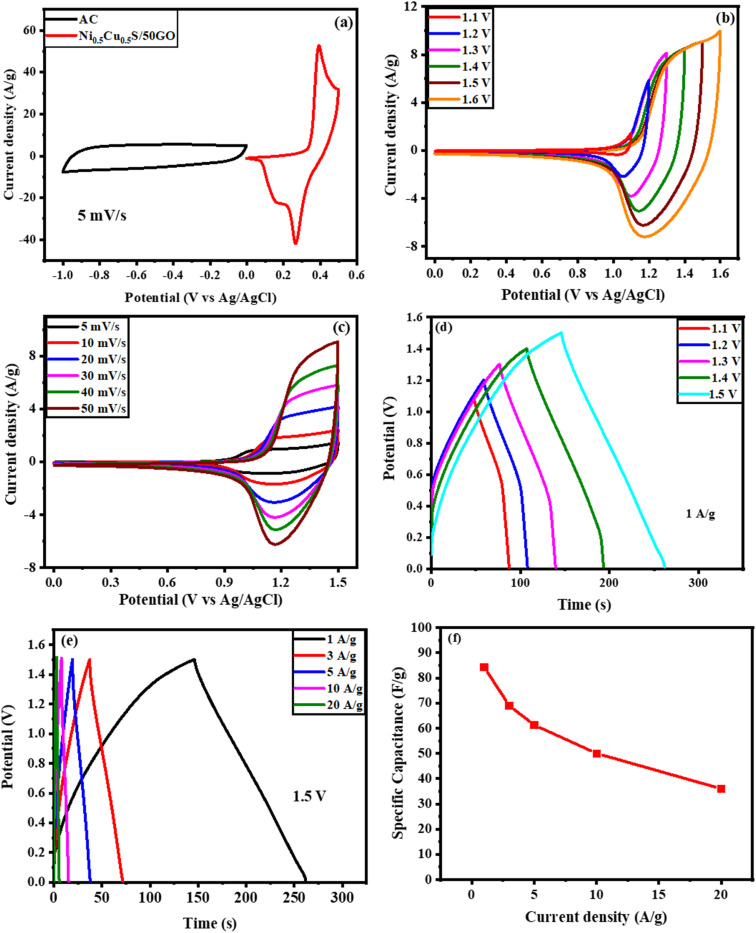
Electrochemical performance of Ni_0.5_Cu_0.5_S/50rGO/NF//AC device; (a) CV curves of AC and Ni_0.5_Cu_0.5_S/50rGO (5 cycles) in three-electrode configuration at 5 mV s^−1^, (b) CV curves at a scan rate of 50 mV s^−1^ through several voltage windows, (c) CV curves as a function of scan rate at (0–1.5 V voltage window), GCD curves (d) within different voltage windows and (e) as a function of current density, and (f) the measured capacitance at different current densities.

An asymmetric hybrid supercapacitor device Ni_0.5_Cu_0.5_S/50rGO/NF//AC was constructed using the Ni_0.5_Cu_0.5_S/50rGO as an anode and active carbon as a cathode to further examine the Ni_0.5_Cu_0.5_S/50rGO electrode for potential practical applications. The comparison CV curves of Ni_0.5_Cu_0.5_S/50rGO and activated carbon are presented in Fig. 9a at three electrode configurations at a scan rate of 5 mV s^−1^. The CV curve of Ni_0.5_Cu_0.5_S/50rGO shows a redox peak, and the CV curve of AC indicates a rectangle-like shape, suggesting the different energy storage mechanisms of pseudocapacitance materials and double-layer materials. At a scan rate of 50 mV s^−1^, the CV measurements were conducted for the device with several potential windows from 1 to 1.6 V (Fig. 9b). The figure displays that the CV curves do not deform noticeably at all potential windows and do not show the polarization phenomenon below 1.5 V indicating that the potential window of 1.5 V can be preferred for the practical application of the as-fabricated device. Also, gas will be produced as a result of the electrolyte's splitting decomposition at 1.5 V, which is harmful to the device's life and safety.^46^ Moreover, Fig. 9c displays the CV curves as a function of the scan rate for an asymmetric Ni_0.5_Cu_0.5_S/50rGO/NF//AC supercapacitor across 0–1.5 V voltage window, demonstrating the combination of the EDL capacitor performance and faradaic redox process. It was observed that even at high scan rates, the shape of the CV curves of the device was maintained confirming the fast charge/discharge process and hence the high performance of the device. The GCD technique was applied at 1 A g^−1^ at several voltage windows (Fig. 9d). The figure shows that the as-fabricated device achieved its maximum GCD cycle performance at a 1.5 V voltage window. On the other hand, the measured GCD curves as a function of current density (1–20 A g^−1^) showed an almost symmetrical shape and low IR drops (Fig. 9e), implying high electrical conductivity and desirable electrochemical reversibility. The specific capacitances were conducted from GCD measurements and found to be 84.3 and 36 F g^−1^ at 1 A g^−1^ and 20 A g^−1^, respectively. Fig. S11a† shows the Nyquist plot of the Ni_0.5_Cu_0.5_S/50rGO/NF//AC device throughout the fitting of EIS measurements in the frequency range from 0.1 Hz to 10^5^ Hz. Fig. S11b† observes the Bode plot of the device which presents the phase angle value (66°). The relaxation time value is 0.15 s from the Bode plot, as derived from the frequency values (*f*) at phase angle 45°. Finally, Fig. S12c and d† show the plot of real capacitance (*C*′) and the imaginary capacitance (*C*′′) *versus* the frequency. The faradaic behaviour of the device was confirmed by the low resistance of the charge transfer in the high-frequency range while the extremely low internal resistance at low frequencies demonstrated the high diffusion of electrolytes in the device.

**Fig. 10:**
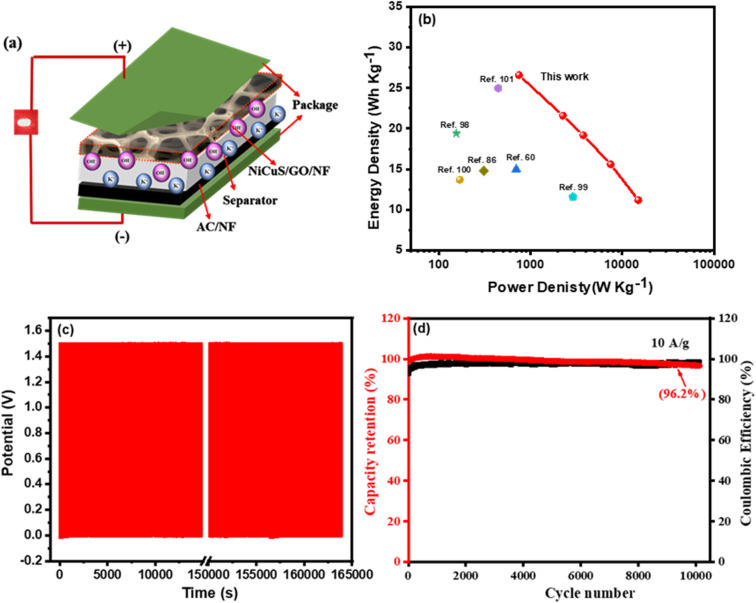
(a) Scheme of the fabricated supercapacitor, (b) the device Ragone plot with a comparison of the calculated power and energy densities with some published works, (c) the device stability from GCD measurements for 1000 cycles, and (d) the capacity retention and coulombic efficiency for the device.

The Ragone plot which is the plot of the calculated energy densities against the calculated power densities for the Ni_0.5_Cu_0.5_S/50rGO (5 cycles)/NF//AC device (Fig. 10a) is shown in Fig. 10b. The device exhibited a high energy density above 26.3 W h kg^−1^ at the 749 W kg^−1^ power density and could still maintain high values of 21.6, 19.2, 15.6, and 11.2 W h kg^−1^, even at the high-power densities of 2254, 3456.5, 7488, and 14 933.3 W kg^−1^, respectively. Additionally, the plot showed a comparison of the Ragone plot of this work with previously reported transition metal sulfide and carbon-based electrodes. Obviously, these values are relatively better than those of Mn–CoP/NF//AC,^86^ NiCoS//AC,^98^ NiCo_2_S_4_//AC,^99^ NiS-rGO//AC,^100^ nitrogen-doped carbon derived (NC)//NC,^60^ and NiS_2_/MoS_2_/rGO//AC.^101^ Finally, the stability of the constructed device was measured for 10 000 cycles (Fig. 10c), and as shown in Fig. 10d, only 6% capacitance loss at 10 A g^−1^ current density and a very high coulombic efficiency of 98.5% after 1000 cycles were noticed. These extraordinary results may be due to the sufficient void spaces between the interconnected Ni_0.5_Cu_0.5_S/50rGO electrode which controls the volume variations, good adhesion to the Ni foam substrate, and superior mechanical stability during the cycling.

## 4. Conclusion

In summary, a facile, low-cost, and one-step electrodeposition strategy was utilized to construct a ternary binder-free Ni_0.5_Cu_0.5_S/rGO electrode on Ni foam. Taking full features of the Ni foam, the fast ion diffusion and pseudocapacity effect of bimetallic sulfides, and the high conductivity of 3D rGO, a binder-free Ni_0.5_Cu_0.5_S/rGO electrode offered an improved electrochemical performance. Therefore, the deposited material displayed a preferable specific capacitance of 2211.029 F g^−1^ at a scan rate of 5 mV s^−1^ and high electrochemical stability for 5000 cycles, even at a high current density which can be related to the fast-charging transportation in the electrode. To examine the practical feasibility of the Ni_0.5_Cu_0.5_S/rGO/NF electrode, an asymmetric supercapacitor that consists of the active carbon/NF as a cathode and the Ni_0.5_Cu_0.5_S/rGO/NF as an anode under the KOH electrolyte has been assembled. The as-synthesized Ni_0.5_Cu_0.5_S/rGO/NF//AC device can achieve an effective specific capacitance of 84.3 F g^−1^ at a 1.5 V potential window and 1 A g^−1^ current density. Besides, after 10 000 GCD cycles, an impressive stability of 96.2% and a high energy density of 26.3 W h kg^−1^ at 749 W kg^−1^ power density were achieved. Thus, the produced device demonstrates a broad potential for various energy storage technologies.

## Conflicts of interest

The authors declare no competing financial interest.

## Supplementary Material

RA-013-D3RA05326A-s001
