# Supplementary material for: Construction of thickness-controllable bimetallic sulfides/reduced graphene oxide as a binder-free positive electrode for hybrid supercapacitors
Source: RSC Adv. 2023 Oct 5;13(42):29252–69. doi: 10.1039/d3ra05326a (PMC10551804; doi:10.1039/d3ra05326a)
Supplement: RA-013-D3RA05326A-s001 [file RA-013-D3RA05326A-s001.pdf]

Electronic Supplementary Material (ESI) for RSC Advances.
This journal is © The Royal Society of Chemistry 2023

Table of Contents Entry

Construction of thickness-controllable bimetallic sulfides/reduced graphene oxide as a binder-free positive electrode for hybride supercapacitors

Ramage M. Ghanem[a], Doaa A. Kospa[a], Awad I. Ahmed[a], Amr Awad Ibrahim[a]*, Ahmed Gebreil[b]

[a]*Department of Chemistry, Faculty of Science, Mansoura University, Al-Mansoura 35516, Egypt.*

[b] *Nile Higher Institutes of Engineering and Technology, El-Mansoura, Egypt.*

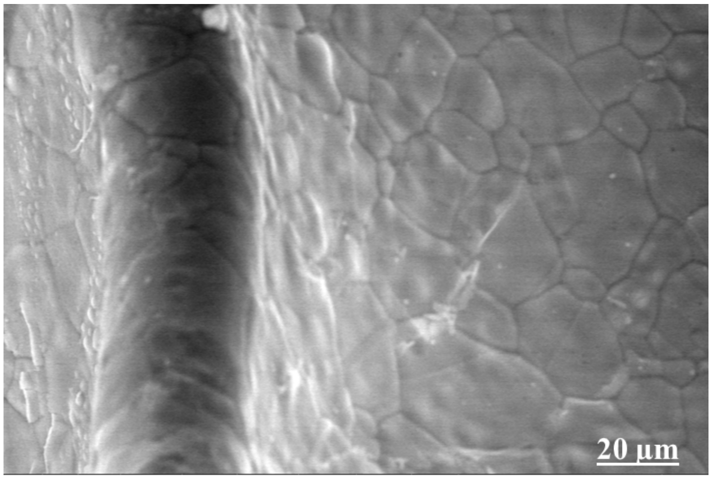

Fig. S1: SEM images of NiCuS/5rGO/NF deposited at controllable CV cycles.

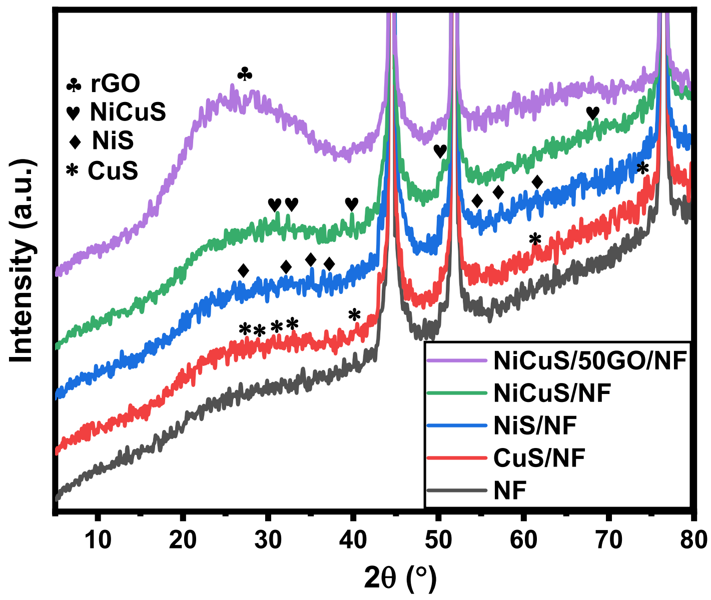

Fig. S2: FT-IR spectra of the as-synthesized materials.

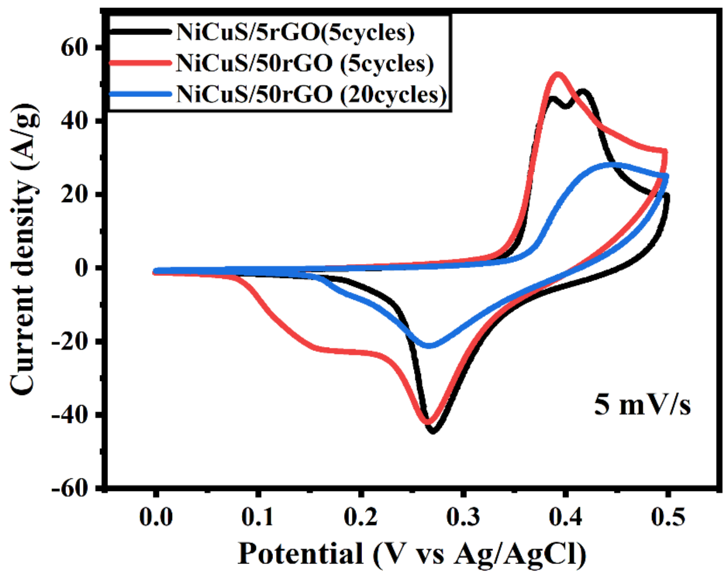

Fig. S3: CV of NiCuS/5rGO and NiCuS/50rGO (5 cycles) and NiCuS/50rGO (20 cycles) at scan rate 5mV/s.

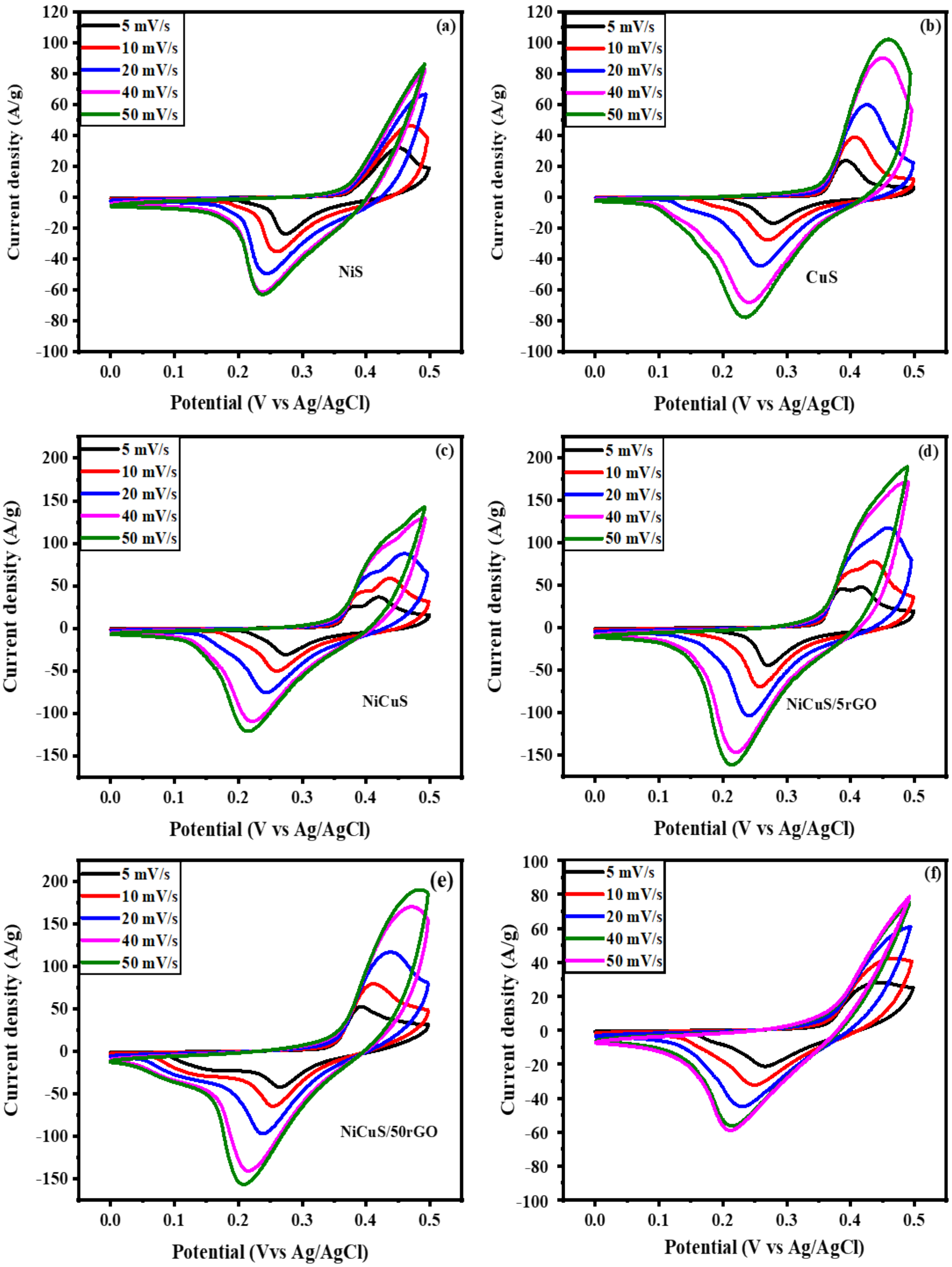

Fig. S4: The CV curves of (a) NiS, (b) CuS, (c) NiCuS, (d) NiCuS/5rGO, (e) NiCuS/50rGO (5 cycles), and (f) NiCuS/50rGO (20 cycles) electrodes in the range of 0.0V-0.5V at different scan rates.

Determination of Electrochemically Active Surface Area (ECSA) of electrocatalysts

The electrical double layer (EDL) capacitance was used to assess the ECSA of the deposition electrodes. In a limited potential window, all electrode CV curves at various scan rates were recorded, as illustrated in S4. The capacitive current (i_c) should be in direct proportion to the scan rate (v): [1]

$$i_c = vC_{EDL} \qquad (1)$$

where C_{EDL} represents EDL capacitance. Assuming the areal EDL capacitance of carbon (C*) is 13 μF/cm^2 as reported by Ji et al.

To calculate the ECSA, the following equation was applied:

$$ECSA = C_{EDL}/C^* \qquad (2)$$

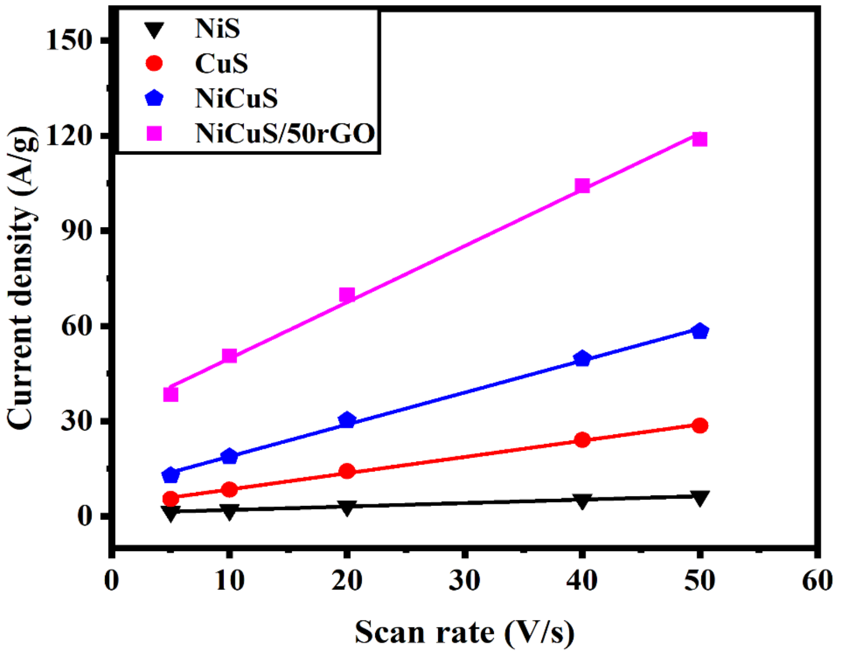

Fig. S5: Capacitive current- scan rate plot of all catalysts.

Table. S1: Comparison of previous reports on metal sulfides for supercapacitors with the fabricated NiCuS/rGO.

Material	Synthesis	Electrolyte	Capacitance	Condition	Retention	No. of Cycle	Ref.
**$NiCu_2S_2$/NF**	Cathodic vacuum arc technique	2 M KOH	1975.2 C/g	0.5 mA/cm^2	76.8% at 10 mA/cm^2	3000	[2]
**NiCu/NF**	Cathodic vacuum arc technique	2 M KOH	739.6 C/g	0.5 mA/cm^2	48.3% at 10 mA/cm^2	3000	[2]
**$Ni_{0.8}Cu_{0.2}S$/CC**	Hydrothermal	2 M KOH	938.6 F/g	1 A/g	69 % at 2 A/g	10000	[3]
**NiCuS/NF**	Hydrothermal	3 M KOH	2.14 F/cm^2	1mA/cm^2	72.2% at 50 mA/cm^2	10000	[4]
**NiCoS/GO/NF**	situ chemical transformation	6 M KOH	1492 F/g	1 A/g	96% at 6 A/g	8000	[5]
**CuMnS//AC**	electrodeposition	1 M KOH	1691 F/g	10 A/g	94% at 20 A/g	2500	[6]
**Annealed-Co_3O_4**	Electrodepostion	1 M KOH	621F/g	5 mA/cm^2	91.4% at 5 mA/g	4000	[7]
**2D- ZnS/FeS @carbon cloth (CC)**	Hydrothermal	6 M KOH	1367.5 F/g	3 A/g	87% at 15 A/g	5000	[8]
**CoS_2-rGO//N-CNT**	Hydrothermal	1 M KOH	1417 F/g	2 A/g	92% at 10 A/g	5000	[9]
**$NiMn_2O_4$@CoS// SCG**	electrodeposition	1 M KOH	1727 F/g	1 A/g	94 % at 10 A/g	5000	[10]
**CuS-NHS (nano hollow sphere)**	hydrothermal method	6 M KOH	948 F/g	1 A/g	90.9% at 2 A/g	1000	[11]
**NiS/GO**	hydrothermal method	2 M KOH	905.3 F/g	0.5 A/g	90.9% at 4 A/g	2000	[12]
**CuS Nanorods**	hydrothermal method	6 M KOH	179 F/g	1 A/g	41% at 2 A/g	1000	[11]
**NiCuS/50rGO//AC**	**Electrodeposition**	**1 M KOH**	**920.1 C/g**	**1 A/g**	**96.2% at 10 A/g**	**10000**	**This work**

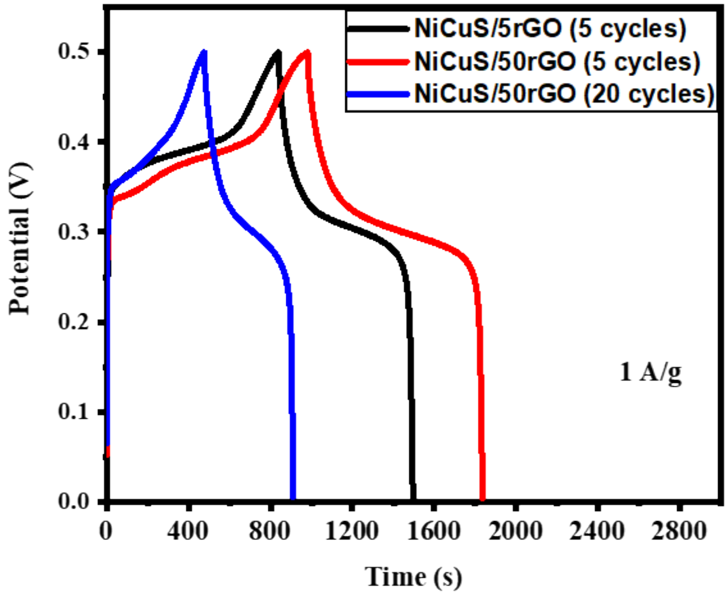

Figure S6: Comparison of the GCD curves of NiCuS/5rGO (5 cycles), and NiCuS/50rGO (5 and 20cycles).

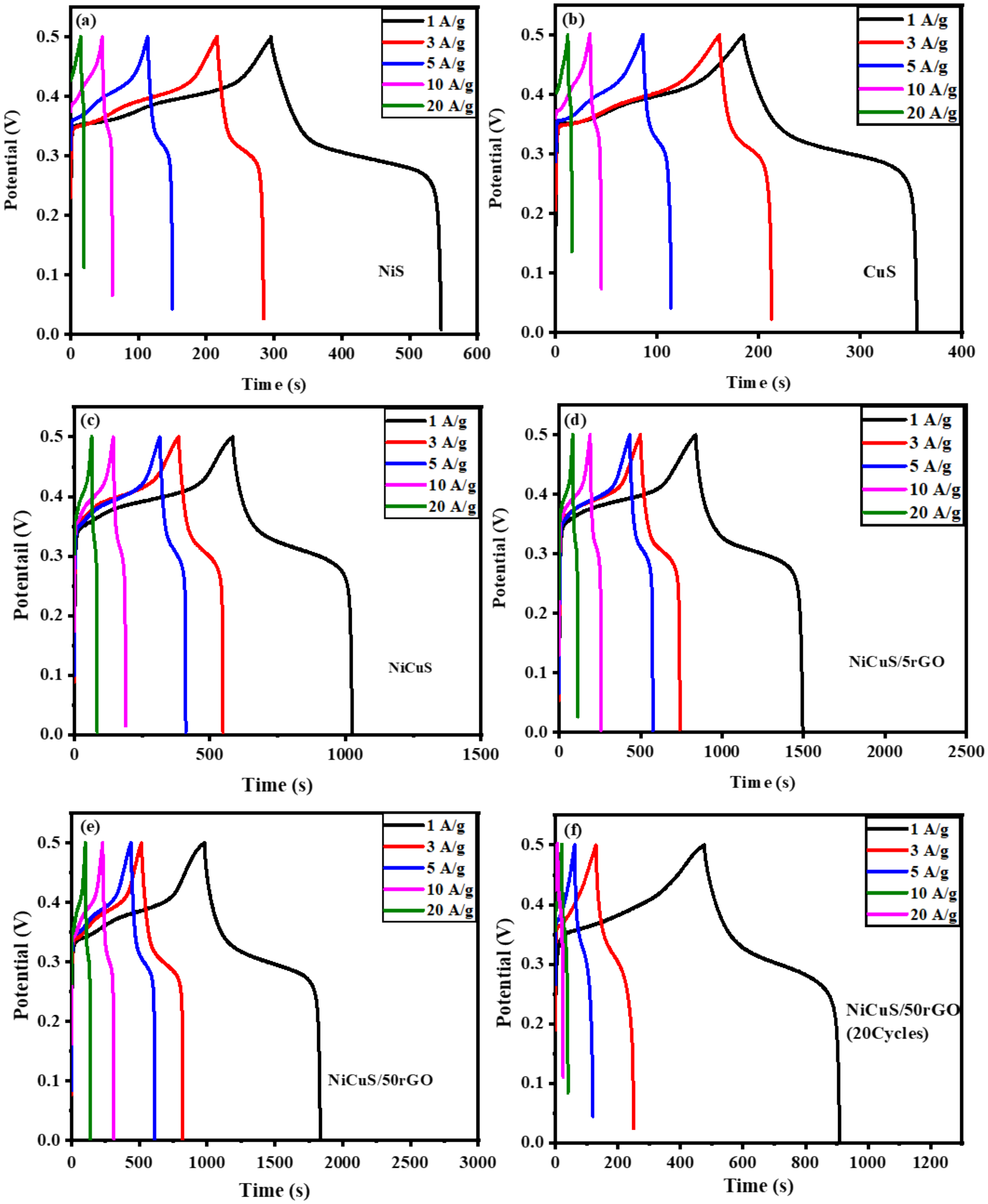

Figure S7: GCD curves of (a) NiS, (b) CuS, (c) NiCuS, (d) NiCuS/5rGO, (e) NiCuS/50rGO (5 cycles), and (f) NiCuS/50rGO (20 cycles) at different current densities.

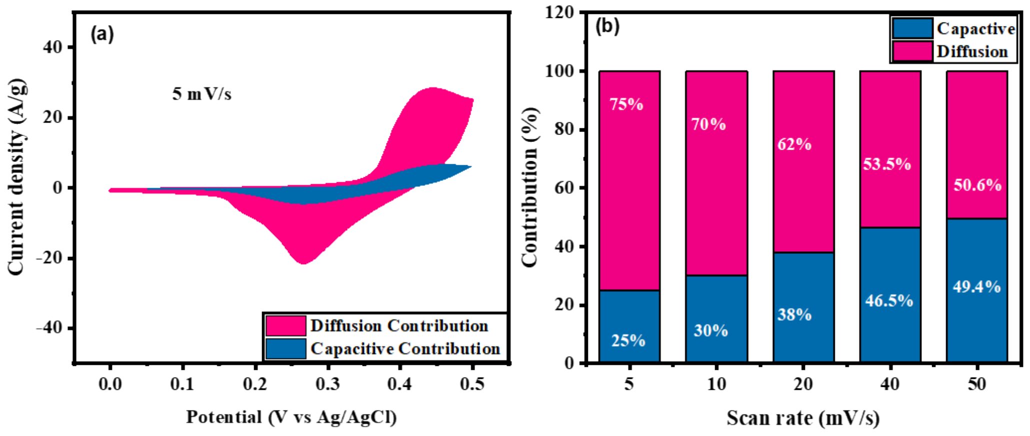

Fig. S8: (a) CV plots of diffusion/ capacitive-controlled contributions at 5mV/s and (b) the ratio of diffusion/ capacitive-controlled contributions as a function of scan rate for NiCuS/50rGO (20 cycles).

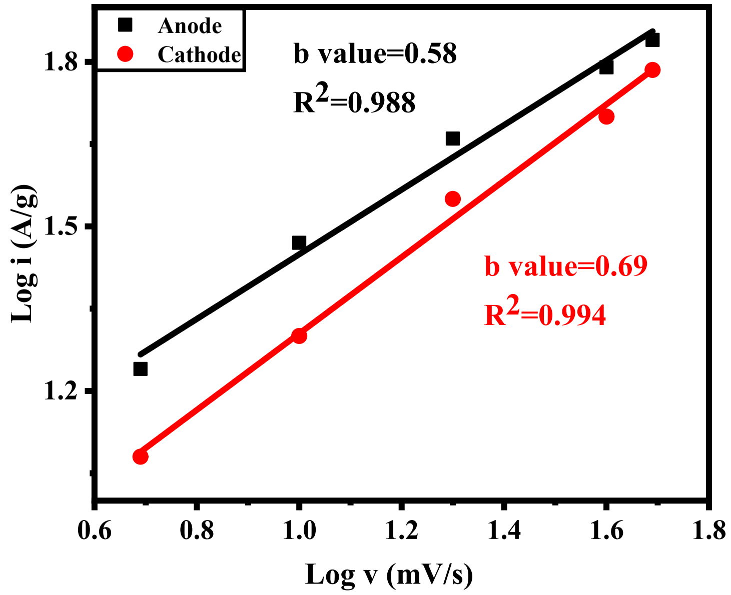

Fig. S9: The relationship between the log (i) and the log(υ) of NiCuS/50rGO (50 cycles).

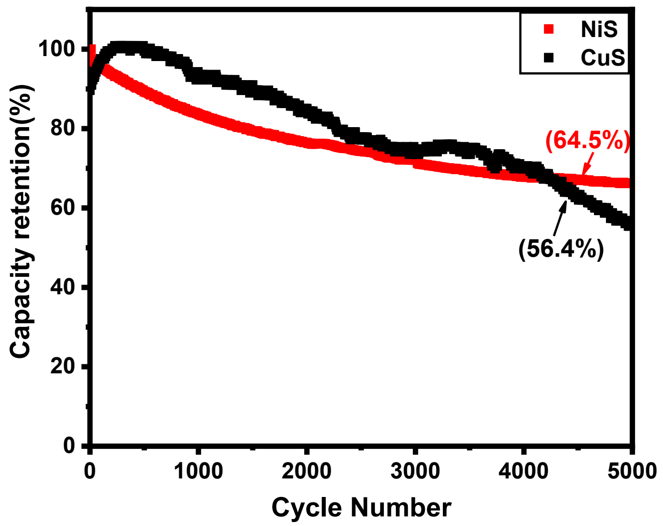

Fig. S10: Cycling stability comparison of NiS and CuS electrodes at 20 A/g.

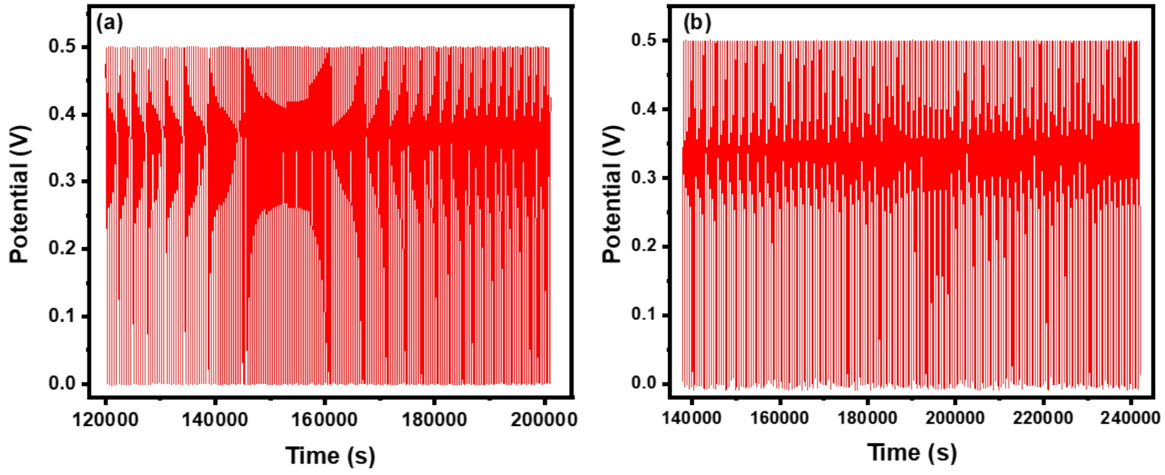

Fig. S11: GCD stability test for (a) NiCuS and (b) NiCuS/50 rGO (5 cycles) for 5000 cycles at 20 A/g current density.

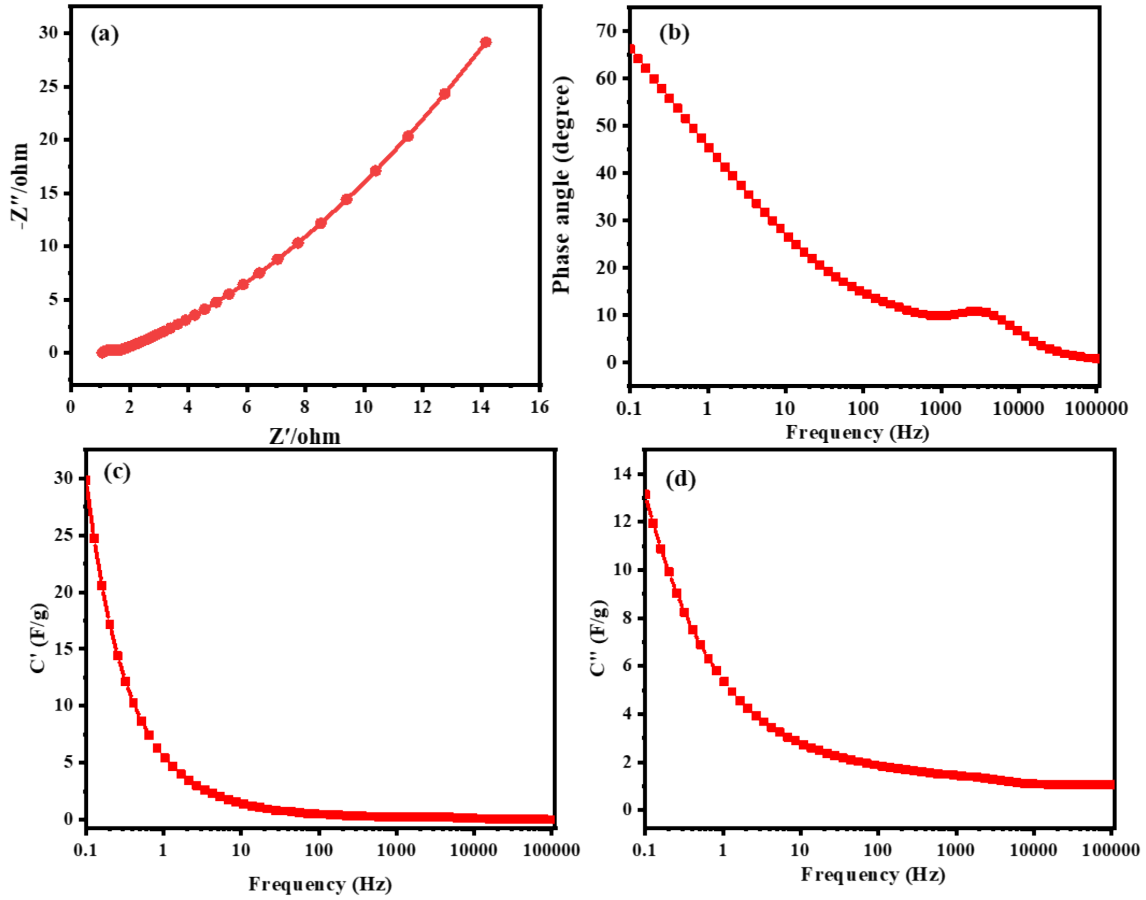

Fig. S12: Nyquist plots of NiCuS/50rGO/NF//AC device from 0.1 Hz to 100 kHz in 0.1 KOH. (a), Bode plots (b), and the real (c) and imaginary (d) capacitances of device versus frequency.

References

1. F. Zhang, H. Liu, Z. Wu, J. Zhang, E. Cui, L. Yue and G. Hou, *ACS Appl. Energy Mater.*, 2021, **4**, 6719-6729.
2. Y. Cheng, M. Zhai and J. Hu, *Appl Surf Sci*, 2019, **480**, 505-513.
3. D. Du, R. Lan, J. Humphreys, H. Amari and S. Tao, *Electrochim Acta*, 2018, **273**, 170-180.
4. X. Xun, H. Liu, Y. Su, J. Zhang, J. Niu, H. Zhao, G. Zhao, Y. Liu and G. Li, *J Solid State Chem*, 2019, **275**, 95-102.
5. J. Yang, C. Yu, X. Fan, S. Liang, S. Li, H. Huang, Z. Ling, C. Hao and J. Qiu, *Energy Environ. Sci.*, 2016, **9**, 1299-1307.
6. M. Z. Iqbal, S. Zakar, S. S. Haider, A. M. Afzal, M. J. Iqbal, M. A. Kamran and A. Numan, *Ceramics International*, 2020, **46**, 21343-21350.
7. M. Kalyani and R. N. Emerson, *J Mater Sci: Mater Electron*, 2019, **30**, 1214-1226.
8. M. S. Javed, T. Najam, M. Sajjad, S. S. A. Shah, I. Hussain, M. Idrees, M. Imran, M. A. Assiri and S. H. Siyal, *Energy Fuels*, 2021, **35**, 15185-15191.
9. B. Hu, H. Li, A. Liu, C. Yue, Z. Guo, J. Mu, X. Zhang and H. Che, *ACS Appl. Energy Mater.*, 2021, **4**, 88-97.
10. N. Hu, L. Huang, W. Gong and P. K. Shen, *ACS Sustain Chem Eng*, 2018, **6**, 16933-16940.
11. H. Heydari, S. E. Moosavifard, S. Elyasi and M. Shahraki, *Appl Surf Sci*, 2017, **394**, 425-430.
12. J. Yang, X. Duan, W. Guo, D. Li, H. Zhang and W. Zheng, *Nano Energy*, 2014, **5**, 74-81.
